# A bispecific, crosslinking lectibody activates cytotoxic T cells and induces cancer cell death

**DOI:** 10.1186/s12967-022-03794-w

**Published:** 2022-12-09

**Authors:** Francesca Rosato, Rajeev Pasupuleti, Jana Tomisch, Ana Valeria Meléndez, Dajana Kolanovic, Olga N. Makshakova, Birgit Wiltschi, Winfried Römer

**Affiliations:** 1 Faculty of Biology, University of Freiburg, Freiburg, Germany; 2 Signalling Research Centres BIOSS and CIBSS, University of Freiburg, Freiburg, Germany; 3 ACIB - The Austrian Centre of Industrial Biotechnology, Graz, Austria; 4 Institute of Molecular Biotechnology, Graz University of Technology, Graz, Austria; 5 Spemann Graduate School of Biology and Medicine, University of Freiburg, Freiburg, Germany; 6 Kazan Institute for Biochemistry and Biophysics, FRC Kazan Scientific Center of RAS, Kazan, Russian Federation; 7 Institute of Bioprocess Science and Engineering, University of Natural Resources and Life Sciences, Vienna, Austria; 8 Freiburg Institute for Advanced Studies (FRIAS), University of Freiburg, Freiburg, Germany

**Keywords:** Lectins, Tumor-associated carbohydrate antigens, Bispecific targeting, Globotriaosylceramide, Click chemistry, Cancer immunotherapy, T cells, Shiga toxin

## Abstract

**Background:**

Aberrant glycosylation patterns play a crucial role in the development of cancer cells as they promote tumor growth and aggressiveness. Lectins recognize carbohydrate antigens attached to proteins and lipids on cell surfaces and represent potential tools for application in cancer diagnostics and therapy. Among the emerging cancer therapies, immunotherapy has become a promising treatment modality for various hematological and solid malignancies. Here we present an approach to redirect the immune system into fighting cancer by targeting altered glycans at the surface of malignant cells. We developed a so-called “lectibody”, a bispecific construct composed of a lectin linked to an antibody fragment. This lectibody is inspired by bispecific T cell engager (BiTEs) antibodies that recruit cytotoxic T lymphocytes (CTLs) while simultaneously binding to tumor-associated antigens (TAAs) on cancer cells. The tumor-related glycosphingolipid globotriaosylceramide (Gb3) represents the target of this proof-of-concept study. It is recognized with high selectivity by the B-subunit of the pathogen-derived Shiga toxin, presenting opportunities for clinical development.

**Methods:**

The lectibody was realized by conjugating an anti-CD3 single-chain antibody fragment to the B-subunit of Shiga toxin to target Gb3^+^ cancer cells. The reactive non-canonical amino acid azidolysine (AzK) was inserted at predefined single positions in both proteins. The azido groups were functionalized by bioorthogonal conjugation with individual linkers that facilitated selective coupling via an alternative bioorthogonal click chemistry reaction. In vitro cell-based assays were conducted to evaluate the antitumoral activity of the lectibody. CTLs, Burkitt´s lymphoma-derived cells and colorectal adenocarcinoma cell lines were screened in flow cytometry and cytotoxicity assays for activation and lysis, respectively.

**Results:**

This proof-of-concept study demonstrates that the lectibody activates T cells for their cytotoxic signaling, redirecting CTLs´ cytotoxicity in a highly selective manner and resulting in nearly complete tumor cell lysis—up to 93%—of Gb3^+^ tumor cells in vitro.

**Conclusions:**

This research highlights the potential of lectins in targeting certain tumors, with an opportunity for new cancer treatments. When considering a combinatorial strategy, lectin-based platforms of this type offer the possibility to target glycan epitopes on tumor cells and boost the efficacy of current therapies, providing an additional strategy for tumor eradication and improving patient outcomes.

**Supplementary Information:**

The online version contains supplementary material available at 10.1186/s12967-022-03794-w.

## Background

Cancer is a leading cause of death worldwide, and according to the World Health Organization (WHO) it accounted for nearly 10 million deaths in 2020 [1]. Genetic and epigenetic alterations are recognized as primary causes of cancer development, where changes at the protein level drive cancer progression and dissemination [2]. Moreover, in the past decade glycobiology has gained increased importance in cancer research given its role in understanding cancer mechanisms and shining a new light on potential targets for diagnostic application and therapeutic strategies [3, 4]. Alterations in post-translational modifications such as glycosylation are indeed a common hallmark of cancer cells [5]. Cancer-associated glycans either occur as increased or incomplete branched-structures, appear as neoantigens, or as overexpressed or completely absent glycans [6, 7]. When cells evolve progressively to a neoplastic state, aberrant glycosylation of glycoproteins, glycosphingolipids and proteoglycans plays a crucial role in progression and invasiveness. These tumor-associated carbohydrate antigens (TACAs) contribute to proliferative signaling, the evasion of growth suppressors, resistance to cell death, angiogenesis, and the onset of cancer metastasis, leading ultimately to malignant phenotypes [5, 8]. Therefore, glycans have major potential applications in improving early diagnosis and determination of prognosis, as well as in serving as specific therapeutic targets. Novel strategies have been developed that focus on glycosylation patterns encountered in several cancers, with a major impact on emerging therapies [9].

Several TACAs consist of altered glycosphingolipids (GSLs) [10]. Among them, the glycosphingolipid Gb3 (α-D-Gal(1 → 4)β-D-Gal(1 → 4)β-D-Glc(1 → O-ceramide), also known as CD77 or P^k^ antigen, has been reported to be overexpressed in Burkitt’s lymphoma [11, 12], breast [13, 14], ovarian [15], colorectal [16, 17], and pancreatic cancer [18, 19]. Gb3 is present in the extracellular leaflet of the plasma membrane [20] and is mainly located in lipid rafts, which are membrane domains enriched in sphingomyelin and cholesterol. The ceramide backbone of this GSL is linked to a neutral trisaccharide composed of galactose (Gal) and glucose (Glc) [21]. Its cell surface-exposed oligosaccharide chains have been described as attachment sites for pathogens [22–24]. This receptor is exploited by different bacterial lectins/toxins [25], including Shiga toxins (Stxs) [26], for surface binding [27, 28] and uptake [29], intracellular trafficking, and signaling events [30–32]. Stxs (also termed verotoxins) are produced by *Shigella dysenteriae* serotype 1 and enterohaemorragic *Escherichia coli* (EHEC) strains [33–36] and belong to the AB_5_ family of bacterial toxins. Additionally, some *E. coli* strains produce a second type of Stx, named Stx2, that has the same receptor and mode of action as Stx1, but is immunologically distinct and shares only 56% identity at the amino acid sequence level [37]. Stxs consist of an enzymatically active A-subunit that possesses *N*-glycosidase activity and inhibits protein biosynthesis by modifying host ribosomal RNA (rRNA), and a non-toxic, low immunogenic homopentameric B-subunit [38, 39]. The B-pentamer binds to its preferential globotriaosylceramide with high specificity and in a multivalent fashion [40, 41], presenting up to 15 binding sites for the receptor [42]. Upon binding to Gb3, StxB induces the formation of StxB-Gb3 cluster domains and imposes negative curvature on the host membrane, ultimately leading to lipid reorganization and the formation of narrow tubular membrane invaginations in cells and model membranes [40, 41]. Due to Gb3 implication in human cancers, Stx has found numerous applications as cytotoxic agent or carrier for cytotoxic drugs in cancer treatment [43]. In particular, Shiga toxin 1 B-subunit (Stx1B) has been coupled to several chemotherapeutic compounds for targeting tumors, with excellent outcomes in intracellular transport of such drugs and elimination of cancer cells [18, 19, 44–46]. The efficacy of these approaches suggests that Stx1B could be considered a promising tool for the selective targeting of carcinomas and lymphomas in which Gb3 is a TACA [11–15, 17, 19, 35, 45].

The specificity of several lectins, like the abovementioned Stx1B, towards carbohydrate antigens promotes their applications in biological and therapeutic research. In recent years, lectins have been investigated for a variety of novel medical approaches, including cancer diagnosis, imaging, targeted drug delivery and cancer treatment [47, 48]. By recognizing and binding mono- or oligosaccharides attached to proteins and lipids, lectins can be used to highlight and target precise distinctions in glycan structure or composition in the evolution of diseases [49, 50]. In cancer, cell surface alterations in glycan synthesis and expression constitute targets for lectin-based diagnosis and therapy. In such circumstances, lectins represent an opportunity to complement therapies based on glycan-binding antibodies targeting TACAs on malignant cells, as the development of such antibodies has been promising, but challenging. Indeed, in the past few decades few molecules have been evaluated in preclinical models and have progressed to clinical trials [51–57]. Antibodies represent an attractive class of therapeutics in the fast-developing field of immunotherapy. Remarkably, a large portion of novel treatments that aim at boosting the patients´ immune system in fighting cancer are based on monoclonal antibodies (mAbs), antibody–drug conjugates (ADCs), and bispecific antibodies (bsAbs) [55, 58]. In the mentioned examples, the anti-tumor activity of antibodies has been increased by engineering strategies that improve specific tumor-associated antigen (TAA) engagement. Of particular interest is the creation of bispecific antibodies that possess dual affinities for simultaneous recognition of distinct antigens. An attractive approach is represented by bispecific T cell engager (BiTE) molecules that target the CD3 receptor on T cells and a TAA on cancer cells, at the same time. Since Blinatumomab (MT103), the first CD19/CD3 BiTE approved by the United States Food and Drug Administration (FDA) in December 2014 for clinical use in patients with relapsed and/or refractory (R/R) non-Hodgkin lymphoma and R/R B cell precursor acute lymphoblastic leukemia (B-precursor ALL), this antibody format has seen a rapid development for the treatment of several malignancies [59]. So far, next-generation BiTEs against CD19, EpCAM or EGFR have faced clinical trials [60]. In the design of such antibodies, a CD3-targeted antibody fragment and a tumor antigen-targeted antibody are genetically linked, rendering it possible to activate a T cell when it physically engages a tumor cell and redirects its cytotoxic activity to achieve tumor cell lysis. The concept of retargeting T cell cytotoxicity for cancer therapy goes back to the 1970s, as these cells possess optimal therapeutic features for fighting cancer [61]. T cells are indeed prone to rapidly expand upon activation and can be usually found in high numbers. They elicit strong cytotoxic responses and are able to attack tumor cells. Remarkably low doses of BiTEs can induce anti-tumor activity, and their efficacy is not affected by mutations of downstream signaling pathways that lead, for example, to resistance to monoclonal antibody-based treatments. In fact, T cell-engaging BiTE antibodies do not rely on the inhibition of TAA-induced signaling but use the TAA as surface anchor for attachment of cytotoxic T cells. As such, they bypass mutations in the cancer cells’ signaling components, such as hyper activation of PI3-kinase and loss of PTEN for example [62, 63]. In view of the aberrant expression of certain GSLs in cancer, over the past two decades few antibodies have been evaluated in preclinical studies or clinical investigation. Examples include the mAb hu14.18K322A, which specifically recognizes the ganglioside GD2, evaluated in a phase II trial in neuroblastoma patients [64] and the mAb BIW-8962 against the ganglioside GM2, highly expressed in lung cancer [65]. However, glycans are considered poor immunogens [66, 67] and the challenging generation of high affinity antibodies against TACAs poses a limit to immunotherapy. For this reason, novel therapeutic approaches which employ recognition components other than antibodies are of particular interest.

In the present study, we successfully generated a bispecific T cell engager that replaces the tumor-targeted antibody fragment with a lectin to recognize the tumor-related antigen Gb3. The single chain variable fragment (scFv) OKT3 was chosen as an anti-CD3-binding module [68, 69]. On the other end, the pentameric non-toxic B-subunit of the pathogen-derived Stx1 was selected for the targeting of Gb3-expressing malignant cells. The two proteins were genetically engineered to incorporate the non-canonical amino acid (ncAA) N6-((2-azidoethoxy)carbonyl)-l-lysine (AzK) at predefined permissive positions in their protein sequence. We used strain-promoted azide-alkyne cycloaddition (SPAAC) to functionalize the azido groups with linkers carrying compatible bioorthogonal groups that facilitated the conjugation of scFv OKT3 and Stx1B by inverse electron demand Diels–Alder (IEDDA) reaction. The conjugated product Stx1B-scFv OKT3 was denominated “lectibody”, based on its bispecific composition of a lectin and an antibody scFv. In vitro cell-based assays revealed that the Stx1B-scFv OKT3 lectibody can simultaneously engage CTLs and Gb3^+^ tumor cells, redirecting T cell cytotoxicity in a highly selective manner and resulting in nearly complete tumor cell lysis. This lectibody format shows promising features in targeting tumor-associated glycans and represents a new modality to complement existing cancer therapies.

## Material and methods

### Antibodies and chemicals

The following antibodies from BioLegend (San Diego, CA, USA) were used: biotin-labeled anti-human CD3 (UCHT1) (Cat. No. 300404), Alexa Fluor^®^ 647 anti-human CD3 (OKT3) (Cat. No. 317312), FITC anti-human CD3 (OKT3) (Cat. No. 317306), Pacific Blue™-labeled anti-hCD8 (Cat. No. 344718), APC-conjugated anti-hCD69 (Cat. No. 310909), FITC-conjugated anti-hCD71 (Cat. No. 334103), APC-labeled anti-hCD25 (Cat. No. 985810), Alexa Fluor 647-labeled anti-6-His epitope tag (Cat. No. 362611), FITC-conjugated anti-6-His epitope tag (Cat. No. 362618).

The following were obtained from commercial sources: RPMI 1640, DMEM, PBS, HEPES, FBS, L-glutamine and 0.05% Trypsin–EDTA (1x) were purchased from Gibco (Thermo Fisher Scientific Inc., Rockford, IL, USA). DMSO, penicillin/streptomycin, ß-mercaptoethanol, isopropyl-ß-d-1-thiogalactopyranoside (IPTG), Luria Bertani (LB) agar and Luria Bertani broth were obtained from Carl Roth GmbH & Co. (KG, Germany). d-Luciferin Firefly was provided by Biosynth (Staad, Switzerland), Pancoll, DMEM (w: 1.0 g/L Glucose, w: l-Glutamine, w: Sodium pyuvate, w: 3.7 g/L NaHCO_3_) and MEM NEAA (Non Essential Amino Acid Solution 100x) were purchased from PAN Biotech (Bayern, Germany). DL-threo-1-phenyl-2-palmitoylamino-3-morpholino-1-propanol (PPMP) was obtained from Sigma-Aldrich Chemie GmbH (Germany). All restriction enzymes (FastDigest) were purchased from Thermo Fisher Scientific (Waltham, MA, USA). The Wizard^®^ SV Gel and PCR Clean-Up System and Wizard® *Plus* SV Minipreps DNA Purification kit were obtained from Promega (Madison, WI).

### Construction of expression vectors

Plasmids encoding Stx1B and Stx1B K9AzK were based on our previously published pT7 × 3 vector [70] with slight modifications. The Stx1B insert was produced by splicing two DNA fragments with overlapping sequences produced in two PCR steps. First, the Stx1B coding sequence with a C-terminal 6× histidine-tag was PCR amplified with primers 2609 F (5′-CATATGACGCCTGATTGTGTAACTGG-3′; NdeI site underlined) and 2607 R (5′-GAAGATCTTTATTAGTGATGGTGATGGTGATGGCCAG-3′; BglII site underlined) using an in-house plasmid [71] as the template. Secondly, a 95 bp sequence immediately upstream of the gene of interest (GOI) coding region in the target vector pT7 × 3 [70] was amplified with primers 2610F (5′-ACCGCTCGAGTAATACGACTCACTATAGGG-3′; XhoI site underlined) and 2608R (5′-CCAGTTACACAATCAGGCGTCATATGTAATTCTCCTTCTTAAAG-3′; NdeI site underlined). The reverse primer 2608R includes an ACT to CAT exchange upstream of the initiation codon (ATG) of the GOI to introduce an *NdeI* recognition site (CATATG) in the target vector p-T7 × 3 and modify it to pSCS-T7 × 31 for faciliated cloning. The two PCR products were spliced by overlap extension PCR following a published procedure [72]. The splice product was resolved on a 0.8% (w/v) agarose gel and purified using the Wizard^®^ SV Gel and PCR Clean-Up System. The purified insert and the target vector pSCS-T7 × 3 were digested with *XhoI/BglII* and ligated. Chemically competent *Escherichia coli* Top10 F′ cells (Thermo Fisher Scientific Inc., Rockford, IL, USA) were heat shock transformed with the ligation mixture, regenerated, and plated on LB agar plates employing standard procedures [73]. Plasmids from selected kanamycin-resistant clones were isolated using the Wizard^®^ *Plus* SV Minipreps DNA Purification kit and were sequenced (Microsynth AG, Balgach, Switzerland) using the 2609 F and 2607 R primers. The expression vector pSCS-T7 × 31-Stx1B K9am-6xH was constructed by PCR amplification of the Stx1B K9am gene from pSCS-Stx1B K9am [71] using primers 2609 F and 2607 R. We replaced the Stx1B coding sequence in pSCS-T7 × 31-Stx1B-6 × H with the Stx1B K9am PCR product by restriction cloning using NdeI/BglII. Chemically competent *E. coli* BL21(DE3) cells (Merck KGaA, Darmstadt, Germany) were transformed with the sequence verified pSCS-T7 × 31-Stx1B-6 × H and pSCS-T7 × 31-Stx1B K9am-6 × H plasmids and stored in 30% (v/v) glycerol at − 80 °C. The construction of plasmids encoding scFv OKT3 and scFv OKT3 E129AzK will be described elsewhere (manuscript in preparation).

### Protein expression

To prepare an overnight pre-culture, 10 mL LB medium containing 50 µg/mL kanamycin (LBkan) were inoculated from the corresponding glycerol stock and were grown overnight (ON) at 37 °C with 120 rpm shaking. 10 mL of ON culture was inoculated into 1-L LBkan. The cultures were incubated at 37 °C, shaking at 150 rpm, until the cell density (D_600_) reached 0.8 for Stx1B and 0.5 for scFv OKT3. Protein expression was induced with 0.3 mM IPTG at 20 °C for 16–19 h, shaking at 180 rpm. For azide-labeled variants, freshly prepared non-canonical amino acid H–L-Lys(EO-N3)-OH * HCl (l-azidolysine or AzK; Iris Biotech, Marktredwitz, Germany) in sterile doubly distilled H_2_O (ddH_2_O) was added to a final concentration of 5 mM at the time of IPTG induction. Cells were harvested at 8000 rpm (JA-10.500 rotor, Beckman Coulter Life Sciences, Indianapolis) for 15 min, and cell pellets stored at − 20 °C. scFv OKT3 and scFv OKT3 E129AzK were purified immediately after harvesting without storing the pellets.

### Protein purification

To purify Stx1B and Stx1B K9AzK, the harvested cell pellets were thawed at room temperature (RT) and resuspended in 60 mL of immobilized metal affinity chromatography (IMAC) binding buffer (20 mM Tris, 300 mM NaCl, 10 mM imidazole; pH 8). Cells were physically disrupted using sonication (Branson Sonifier 250, Emerson Electric, St. Louis, MO, USA) for 3 min on ice. The lysed cell suspension was centrifuged at 21,000 rpm for 20 min. The clarified cell lysate in the supernatant was added onto a Zn^2+^ charged sepharose matrix (Chelating Sepharose^®^ Fast Flow, Cytiva, Marlborough, MA, USA) and the flow-through was collected by gravity. The matrix was washed with 25 mL IMAC wash buffer (20 mM Tris, 300 mM NaCl, 25 mM imidazole; pH 8) to remove non-specifically bound proteins. Column-bound protein was eluted with IMAC elution buffer (20 mM Tris, 300 mM NaCl, 400 mM imidazole; pH 8) and collected in 1 mL fractions. The absorbance of the fractions was measured using a spectrophotometer (Thermo Scientific Inc., Rockford, IL, USA) at 280 nm. The fractions with the highest absorbance were pooled and the buffer was exchanged to phosphate-buffered saline (PBS, 137 mM NaCl, 2.7 mM KCl, 10 mM Na_2_HPO_4_, and 1.8 mM KH_2_PO_4_, pH 7.4) using PD-10 desalting columns (GE Healthcare, Boston, MA, USA) prior to lyophilization. At every step, protein gel samples (PGS) of 12 µL were mixed with 3 µL of 5× reducing sample buffer (250 mM Tris, 20% (v/v) glycerol, 5% SDS, 500 mM dithiothreitol, 0.2% (w/v) bromophenol blue, 2.5% (v/v) ß-2mercapto ethanol) and heated at 95 °C for 5–10 min before analysis by SDS-PAGE. scFv OKT3 and scFv OKT3 E129AzK were purified and analysed as described in [74] with modifications (manuscript in preparation).

### Stx1B and scFv OKT3 labelling

The proteins with a final concentration Stx1B (5 µM), Stx1B K9AzK (5 µM), scFv OKT3 (2 µM) and scFv OKT3 E129AzK (2 µM) were separately mixed with a ten-times excess of dibenzocyclooctyne-sulfo-Cy3 (DBCO-Cy3, Jena Bioscience GmbH) in 10 µL volumes in PBS at 22 °C and incubated with shaking at 600 rpm for 1 h in the dark. 5 XSDS reducing sample buffer was added directly to the sample to stop the SPAAC reaction. For the initial assessment of Gb3 abundance at the surface of treated cells, commercial Stx1B (Sigma-Aldrich Chemie GmbH, Germany) was dissolved at 1 mg/mL in PBS and stored at 4 °C prior to its use. For fluorescence labelling, Cy5 mono-reactive NHS ester (GE Healthcare, Boston, MA, USA) was used. The fluorescent dye was dissolved at a final concentration of 10 mg/mL in water-free DMSO, aliquoted, and stored at − 20 °C before usage according to the manufacturer’s protocol. For the labelling reaction, 100 µL of Stx1B (1 mg/mL) was supplemented with 10 µL of a 1 M NaHCO_3_ (pH 9) solution so that the molar ratio between dye and lectin was 6:1. The labelling mixture was incubated at 25 °C for 60 min under continuous stirring, and uncoupled dyes were removed using Zeba™ Spin desalting columns (7 kDa MWCO, 0.5 mL, Thermo Fischer Inc., Rockford, IL, USA). Cy5-labelled Stx1B was stored at 4 °C with protection from light.

### Conjugation of Stx1B and scFv OKT3

The lyophilized Stx1B K9AzK and scFv OKT3 E129AzK were resuspended in sterile ddH_2_O, and the concentrations were measured spectrophotometerically. The linkers methyltetrazine (Tz)-DBCO and *trans*-cyclooctene (TCO)-PEG4-DBCO from Broadpharm (San Diego, CA) were dissolved in DMSO to a concentration of 100 mM and stored as 10 µL aliquots at − 20 °C in the dark until use. SPAAC was performed at RT for 2 h in 100 µL volumes of PBS (pH 7.4) containing 60 µM Stx1B K9AzK and 600 µM methyltetrazine-DBCO or 20 µM scFv OKT3 E129AzK and 200 µM TCO-PEG4-DBCO. The DMSO concentration was maintained between 8 and 10% (v/v) in the reaction mixtures. To stop the reaction and to remove the unreacted linkers, the reaction mixture was buffer exchanged with PBS (pH 7.4) using 0.5 mL Zeba Spin desalting columns. IEDDA was performed according to [75] by mixing a three-times excess of Stx1B K9Tz to scFv OKT3 E129-PEG4-TCO at RT for 1.5 h. The IEDDA reaction mixture was separated by gel filtration chromatography (see sections below). For SDS-PAGE analysis, an aliquot of the sample was mixed with 5 × SDS reducing sample buffer at a ratio of 1:5.

### Electrophoresis

SDS-PAGE: 4–12% NuPAGE bis–tris mini protein precast polyacrylamide gels (Invitrogen) were run for 40 min at 200 V with NuPAGE MES-SDS running buffer (Thermo Fisher Scientific). The gels were stained with InstantBlue^®^ Coomassie Protein Stain (Abcam plc., UK). Gels with fluorophore-labelled samples were washed three times for 15 min each and observed for fluorescence at 635 nm (G:Box F3 gel doc system, SYNGENE, UK) before staining with InstantBlue^®^ Coomassie Protein stain. Native-PAGE: The SERVA*Gel*™ N Native Starter Kit (Serva Electrophoresis GmbH, Heidelberg) was used to run clear native electrophoresis with the native cathode and anode buffers. Two micrograms of Stx1B K9AzK were mixed with 2 X clear native sample buffer (100 mM NaCl, 100 mM imidazole, 4 mM 6-aminocaproic acid, 2 mM EDTA, 0.02% (w/v) Ponceau S, 20% (v/v) glycerol) and resolved on a 3–12% vertical native gel at 50 V for 10 min, and 200 V for 90 min. The gels were developed by silver staining as described in [76].

### Gel filtration chromatography

Superdex 200 Increase 10/300 GL column (Cytiva) was used for a small-scale preparative purification and characterization of the Stx1B-scFv OKT3 conjugate. The column was connected to an ÄKTA pure chromatography system (GE Healthcare) and calibrated with molecular weight standards aprotinin (6500 Da), ovalbumin (43,000 Da), conalbumin (75,000 Da), aldolase (158,000 Da) and ferritin (440,000 Da) from GE Healthcare. Theoretical molecular weights for scFv OKT3 and STx1B-scFv OKT3 were calculated using Protparam Expasy [77].

### Mass spectrometry

Proteins were desalted using Amicon centrifugal filters before analysis. A volume of 5 µL protein with a concentration ranging between 0.1 and 0.3 mg/mL was injected into a LC–ESI–MS system (LC: Agilent 1290 Infinity II UPLC). A gradient from 15 to 80% acetonitrile in 0.1% (v/v) formic acid [using a Waters BioResolve column (2.1 × 5 mm)] at a flow rate of 400 μL/min was applied (9-min gradient time). A Q-TOF instrument (Agilent Series 6560 LC-IMS-QTOFMS) equipped with the Jetstream ESI source in positive ion, MS mode (range: 100–3200 Da) was used for detection. ESI calibration mixture (Agilent) was used to calibrate the instrument. MassHunter BioConfirm B.08.00 (Agilent) was used for data processing and the spectrum was deconvoluted by MaxEnt.

### Isothermal titration calorimetry

MicroCal PEAQ-ITC (Malvern Panalytical ltd, Malvern, UK) microcalorimeter was used to perform ITC experiments. Lyophilized Stx1B and Stx1B K9AzK were dissolved in sterile ddH_2_O. Buffer was exchanged to PBS (pH 7.4) using PD-10 desalting columns. Globotriose (Gb3) (ELICITYL, Crolles, Frances) was resuspended in the same buffer. Gb3 (50 mM) was titrated into the sample cell with 190 µM Stx1B or Stx1B K9AzK in a total of fourteen 2.8 µL injections, each spaced at 300 s. Data were analysed using a one-site binding model in Microcal origin (OriginLab, Northampton, MA, USA).

### Structural modelling

The structural model of the lectibody was constructed on the base of StxB 3D structure (PDB code 1BOS) and structural information about OKT3 Fab from *Mus musculus* (PDB code 1SY6). The fragments of heavy and light chains of OKT3 Fab that shared high percentage of identity with scFv OKT3 were used as a template for the homology modelling of scFv OKT3. The mutual orientation of domains in the resultant protein was kept the same as in the template. The homology modelling was performed using the Modeller program v9.15 [78]. The StxB and scFv OKT3 were linked via the DBCO-methyltetrazine-DBCO linker. The assembled structure was energy minimized in OPLS2005 force field implemented in Maestro Schrodinger [79] using the Polak-Ribier Conjugate Gradient method [80] with a gradient convergence threshold of 0.05 kJ/mol/Å.

### Cell lines

Human Jurkat T cells (American Type Culture Collection, TIB-152, Thermo Fisher Scientific Inc., Rockford, IL, USA), HEK293T cells, Burkitt´s lymphoma Ramos and Namalwa cell lines, HT-29 and LS-174 colon adenocarcinoma cell lines were used in this study (Ramos cells were kindly provided by Prof. Dr. Michael Reth, Institut für Biologie III, Albert-Ludwigs Universität Freiburg, Germany; NAMALWA.CSN/70, ACC 70, DSMZ—German Collection of Microorganisms and Cell Cultures GmbH; HT-29 and LS-174 cells were kindly provided by PD Dr. Susana Minguet, Institut für Biologie III, Albert-Ludwigs Universität Freiburg, Germany). Ramos, Namalwa, HT-29 and LS-174 cells were transduced with lentiviruses harboring the plasmid pHRSIN-CS-Luc-IRES-emGFP (this plasmid and HEK293T cells were kindly provided by PD Dr. Susana Minguet, Institut für Biologie III, Albert-Ludwigs Universität Freiburg, Germany). Jurkat T cells were maintained in Roswell Park Memorial Institute (RPMI) 1640 medium supplemented with 10% (v/v) heat-inactivated fetal bovine serum (FBS), 2 mM l-glutamine, 2.5 μg/mL penicillin/streptomycin, 0.1% (v/v) of a phosphate-buffered saline (PBS) solution containing ß-mercaptoethanol, 1% (v/v) HEPES in a humidified incubator with 5% CO_2_ at 37 °C. Ramos and Namalwa cells were cultivated in RPMI 1640 medium containing 10% (v/v) FBS, 2 mM l-glutamine and 5 μg/mL penicillin/streptomycin. HT-29 and LS-174 cells were cultivated in Dulbecco's Modified Eagle Medium (DMEM) medium (w: 1.0 g/L Glucose, w: stable Glutamine, w: Sodium pyruvate, w: 3.7 g/L NaHCO3) to which 10% (v/v) FBS, 1% (v/v) HEPES, 1% (v/v) MEM NEAA and 2.5 μg/mL penicillin/streptomycin were added. HEK293T cells were grown in a humidified incubator with 7.5% CO_2_ at 37 °C in DMEM medium complemented with 10% (v/v) FBS, 2.5 μg/mL penicillin/streptomycin, 0.1% (v/v) of PBS solution containing ß-mercaptoethanol, 1% (v/v) sodium pyruvate. If not stated differently, all experiments were performed in the described complete media.

### Lentiviral transduction

A total of 10^7^ HEK293T cells were plated and incubated overnight. After medium exchange, the HEK293T cells were transfected with the packaging plasmid pMD2.G, the gag/pol pCMVR8.74 and the plasmid pHRSIN-CS-Luc-IRES-emGFP (kindly provided by PD Dr. Susana Minguet, Institut für Biologie III, Albert-Ludwigs Universität Freiburg, Germany) using Polyethylenimine (PEI) (Polysciences Inc., Warrington, PA, USA) transfection. The medium containing lentiviral particles was collected at 24 and 48 h post-transfection and concentrated by a 10% sucrose gradient. After 4 h of centrifugation at 10,000 rpm (Rotor FA-45-6-30, Eppendorf SE, Hamburg, Germany) and 6 °C, the supernatant was discarded, and the virus pellet was resuspended in PBS to be stored at − 80 °C. Concentrated lentiviruses were used to transduce Ramos, Namalwa, HT-29 and LS-174 cells by spin infection [81] (multiplicity of infection of 10). Afterwards, B cells and colon adenocarcinoma cells were cultured with the lentiviruses in RPMI 1640 or DMEM medium, respectively, at 37 °C for 48 h.

### Primary human T cells isolation

Peripheral blood mononuclear cells (PBMCs) were isolated from leukoreduction system chambers using density centrifugation (Pancoll human), according to the BioSharing protocol [82]. PBMCs were adjusted to 10^6^ cells/mL and cultured in RPMI 1640 supplemented with 10% (v/v) fetal bovine serum (FBS), 2 mM l-glutamine, 2.5 μg/mL penicillin/streptomycin, 0.5% (v/v) of a PBS solution containing ß-mercaptoethanol, 1% (v/v) HEPES in a humidified incubator with 5% CO_2_ at 37 °C, or frozen in cryovials (20 × 10^6^ cells per vial, in 0.5 mL FBS solution containing 10% (v/v) sterile DMSO) for long-term preservation.

### Depletion of glucosylceramide-based glycosphingolipids by PPMP treatment

To deplete Ramos and HT-29 cells of globotriaosylceramide, 2 × 10^5^ Ramos B cells and 1 × 10^5^ HT-29 cells were seeded in 6-well plates and cultured for 72 h in the presence of 2 μM DL-threo-1-phenyl-2-palmitoylamino-3-morpholino-1-propanol (PPMP), an inhibitor of the synthesis of glucosylceramide-based GSLs [83]. Depletion of globotriaosylceramide from the plasma membrane of treated cells was assessed by flow cytometry analysis by using 2.6 nM Stx1B-Cy5 in binding assays.

### Flow cytometry

For flow cytometry sample preparation, cells were counted and transferred to a U-bottom 96 well plate (Sarstedt AG & Co. KG, Numbrecht, Germany). For binding assays, Ramos, Namalwa and Jurkat T cells were resuspended to a concentration of 1 × 10^5 ^cells/well, while PBMCs were used at a final concentration of 2 × 10^5^ cells/well. HT-29 and LS-174 were detached from culture dish with 2 mL of 0.05% Trypsin–EDTA (1x) solution for 10 min at 37 °C. To quantify binding of wild-type Stx1B, mutant Stx1B K9AzK and lectibody (Stx1B-scFv OKT3) to cell surface receptors, cells were incubated with proteins for 30 min on ice, compared to PBS-treated cells as the negative control. Subsequently, cells were centrifuged at 1600×*g* for 3 min on ice and washed twice with FACS buffer (PBS supplemented with 3% FBS v/v). When Stx1B wt, Stx1B K9AzK or Stx1B-scFv OKT3 produced in this study were applied, cells were stained with fluorescently labelled anti-6-His epitope tag Alexa Fluor 647 antibody diluted in FACS buffer, for 20 min on ice and protected from light. For the characterization of CD3 antigens at the membrane, cells were incubated with anti-human CD3 FITC or anti-human CD3 Alexa Fluor^®^ 647 antibodies (OKT3 clones), for 20 min on ice. At the end of incubation, cells were centrifuged and washed twice as described above. After the last washing step, the cells were resuspended with FACS buffer and transferred to FACS tubes (Kisker Biotech GmbH Co. KG, Steinfurt, Germany). The fluorescence intensity of treated cells was monitored immediately at FACS Gallios (Beckman Coulter Inc., USA) and further analyzed using FlowJo V.10.5.3 (FlowJo LLC, BD).

### Cytotoxicity assay

For the Bioluminescence-based cytotoxicity assay, luciferase-expressing Ramos, Namalwa, HT-29 and LS-174 tumor cells were counted and plated at a concentration of 1.5 × 10^4^ cells in 96-well white flat bottom plates in duplicates. Then, 75 μg/mL of D-firefly luciferin potassium salt was diluted in complete medium and added to the tumor cells. Bioluminescence (BLI) was measured in the luminometer (Tecan infinity M200 Pro) to establish the BLI baseline. Subsequently, PBMCs isolated from healthy donor cells were added at 5:1 effector-to-target (E:T) ratio, and BLI was recorded at several times (4, 8, 24, 48, 56 or 72 h) after incubation at 37 °C. The Stx1B-scFv OKT3 lectibody was added in two different concentrations (7 nM and 35.6 nM) to the samples, as indicated. BLI was measured as relative light units (RLUs). RLU signals from tumor cells cultured with PBMCs cells in absence of lectibody determine spontaneous cell death. RLU signals from cells treated with 2% Triton X-100 indicate maximal cell death. Percent of specific killing was calculated with the following formula: percentage specific killing = 100 × (average spontaneous death RLU − test RLU) / (average spontaneous death RLU − average maximal death RLU).

### CD69, CD71 and CD25 upregulation assay

PBMCs were co-cultured with target cells in a 5:1 E:T ratio in presence or absence of Stx1B-scFv OKT3 lectibody. 3 × 10^5^ PBMCs were counted per well and incubated with 6 × 10^4^ Ramos or Namalwa, as indicated. Cells were treated with 35.6 nM lectibody or left untreated in PBS-containing medium. The plate was incubated at 37 °C and 5% CO_2_ for 24 or 48 h. After incubation, cells were centrifuged at 1600×*g* for 3 min on ice and washed twice with FACS buffer (PBS supplemented with 3% FBS v/v). Cells were stained with fluorescently labelled APC-conjugated anti-hCD69, FITC-conjugated anti-hCD71, APC-labeled anti-hCD25 antibodies diluted in FACS buffer, for 20 min on ice and protected from light. At the end of incubation, cells were centrifuged and washed twice as described above. After the last washing step, the cells were resuspended in FACS buffer and transferred to FACS tubes (Kisker Biotech GmbH Co. KG, Steinfurt, Germany). The fluorescence intensity of treated cells was monitored immediately at FACS Gallios (Beckman Coulter Inc.) and further analyzed using FlowJo V.10.5.3 (FlowJo LLC, BD).

### Statistical analysis

All data in graphs are presented as mean ± standard deviation (SD) and were calculated from the results of independent experiments. Statistical testing was performed with GraphPad Prism 6.01 software and Microsoft Excel 365 using data of ≥ 3 biological replicates. Statistical differences in independent samples were determined with a two-tailed, unpaired t-test. Tests with a *p*-value ≤ 0.05 are considered statistically significant and marked with an asterisk (*). *p*-values ≤ 0.01 are shown as two asterisks (**), and ≤ 0.001 are summarized with three asterisks (***). Non-significant results are indicated with ns.

## Results

### Prediction of the Stx1B-scFv OKT3 structure

**Fig. 1:**
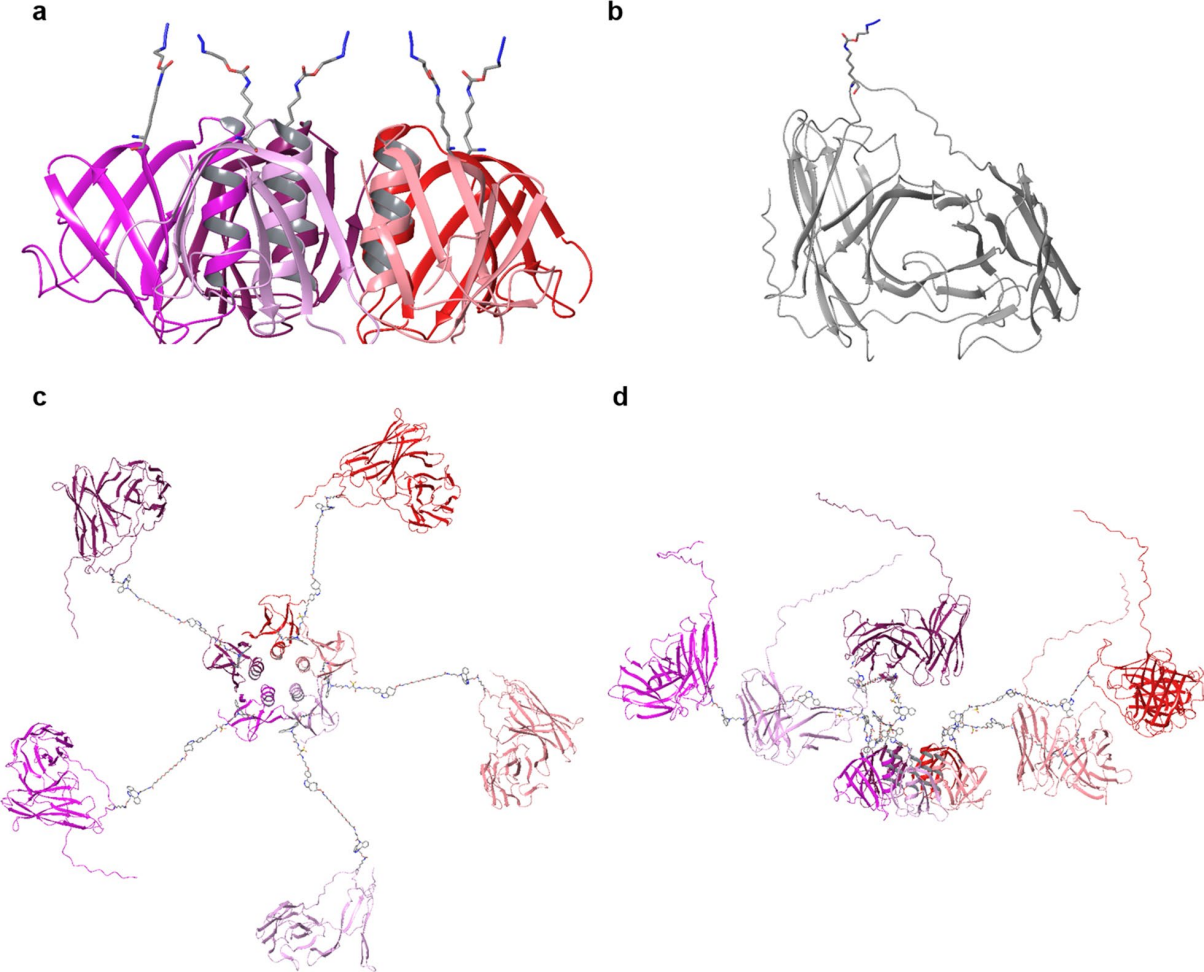
Atomistic models for Stx1B K9AzK, OKT3 E129AzK and Stx1B-scFv OKT3 lectibody. **a** Side view of Stx1B containing AzK at the 9^th^ position in each monomer. The structure is constructed based on PDB:1BOS. The five subunits of Stx1B are shown as red, pink, plum, purple and maroon ribbon cartoons; the AzK are given as sticks with grey color for C atoms, red for O atoms and blue for N atoms, H atoms are not shown for clarity. **b** Structural model of OKT3 E129AzK scFv (AzK is shown in sticks) built using homology modeling based on the template with OKT3 Fab from *Mus musculus* (PDB: 1SY6), the C-terminal fragment with low identity to the template sequence, including 6-His-tag appears as an unstructured tail. **c** Top view and **d** Side view of the Stx1B-scFv OKT3 lectibody model. Each Stx1B monomer is linked to one scFv OKT3 via DBCO-methyltetrazine-DBCO linker connected to the AzK residues. The resulting five subunits of the lectibody are shown in different colors

For the rational design of the lectibody construction, the 3D structures of Stx1B from *Shigella dysenteriae* and the murine hybridoma scFv OKT3 that recognizes an epitope on the human CD3ε subunit of the T cell receptor (TCR) complex were analyzed. The AzK residue was incorporated in protein sequences according to the following conditions: (1) the ncAA should be located at the surface of protein; (2) the residue should be inserted at a distance from the receptor (Gb3 or CD3) binding site, to avoid interference with the binding; (3) the residues in five monomers should be located at a distance from each other to facilitate their derivatization. In the Stx1B sequence, the K9 amino acid, oriented such that it opposes the glycolipid binding pockets facing the membrane (Fig. 1a), suits this purpose perfectly. The residue E129 in the linker connecting the two domains in scFv OKT3 was chosen to site-specifically incorporate AzK (Fig. 1b). The attached linker does not take part in the CD3 recognition, and the amino acid substitution and modification in this region should not destabilize the protein structure nor the receptor binding. Further analysis showed that, at the chosen length of the linkers, the scFv domains do not sterically interfere with each other and all five sites at Stx1B can be theoretically occupied by scFv OKT3 antibody fragments (Fig. 1c, d).

### Construction of the Stx1B-scFv OKT3 conjugate

We chose position K9 of Stx1B for incorporating AzK and subsequent conjugation of scFv OKT3 since this position is surface exposed and distant from the glycan recognition site [71, 84]. The scFv OKT3 sequence included the double mutations E6Q and C105S for soluble expression [74] and a PelB signal sequence for delivery to the periplasm. We employed the pyrrolysyl-tRNA synthetase from *Methanosarcina mazei* with its cognate amber suppressor tRNA_CUA_ (orthogonal *Mm*PylRS/*Mm*tRNA_CUA_ pair [85]) for the site-specific incorporation of AzK [70] in response to in-frame amber codons at positions K9 and E129 of Stx1B and scFv OKT3, respectively. The corresponding wild-type genes were expressed as benchmark controls. Stx1B and Stx1B K9AzK were expressed and purified from the cytosolic soluble protein fraction as described previously [71] while scFv OKT3 and scFv OKT3 E129AzK were produced in the soluble periplasmic fraction. The amber mutants were only expressed when AzK was supplemented in the culture medium (Additional file 1: Fig. S1a), while no expression occurred in its absence (Additional file 1: Fig. S1b), which confirmed the fidelity of the orthogonal *Mm*PylRS/*Mm*tRNA_CUA_ pair. The titres of the purified proteins per litre of culture medium were 3 mg for Stx1B, 2.8 mg for Stx1B K9AzK (93% of the wild-type), 2 mg for scFv OKT3 and 1.4 mg for scFv OKT3 E129AzK (70% of the wild-type).

**Fig. 2:**
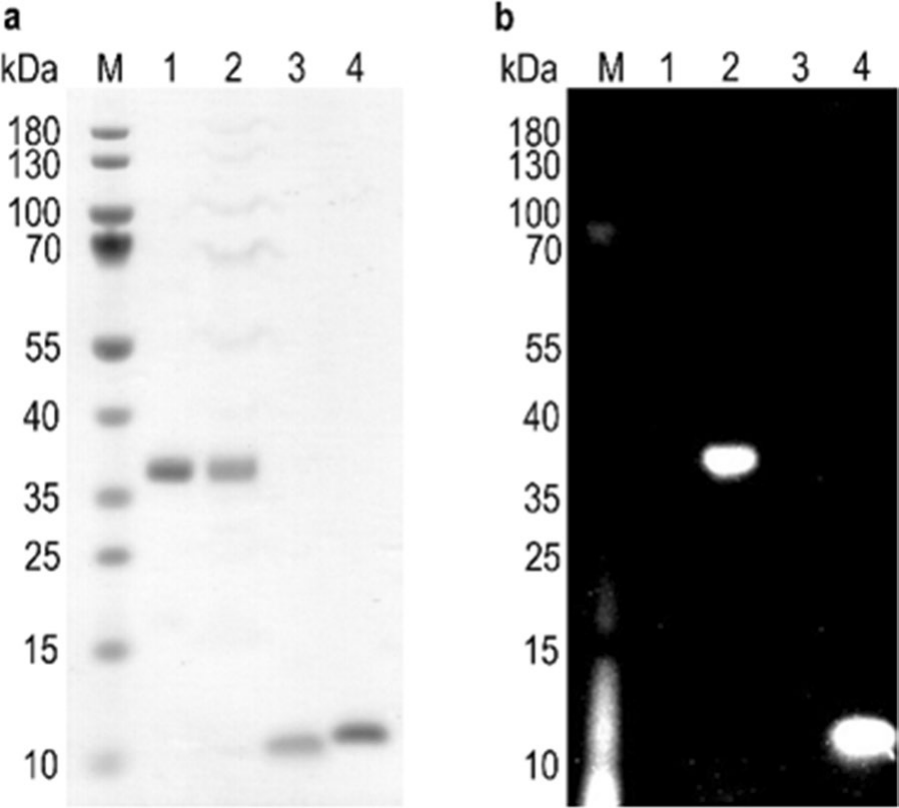
Confirmation of AzK incorporation in Stx1B K9AzK and scFv OKT3 E129AzK by fluorophore labelling. The purified proteins scFv OKT3 (lane 1, MW_calc_ 30 kDa), scFv OKT3 E129AzK (lane 2, MW_calc_ 30 kDa), Stx1B (lane 3, MW_calc_ 9 kDa) and Stx1B K9AzK (lane 4, MW_calc_ 9 kDa) were incubated with DBCO-Cy3 as detailed in the materials and methods section and then analyzed on a 4–12% SDS gel. **a**`Proteins stained with Coomassie protein stain. **b** SDS gel irradiated at 635 nm before staining with Coomassie protein stain. The sizes of the molecular weight marker (M) bands are indicated on the left margin of the gels

We assessed the incorporation of AzK into Stx1B K9AzK and scFv OKT3 E129AzK by labelling the purified proteins with the DBCO-Cy3 fluorophore via SPAAC. The unmodified wild-type proteins were used as a negative control. The proteins containing AzK showed fluorescence at 635 nm indicating Cy3 labelling (Fig. 2), whereas no fluorescence was observed with Stx1B and scFv OKT3 which lacked AzK. This observation confirmed the successful incorporation of AzK into Stx1B and scFv OKT3. Mass analysis of the intact proteins compared with the theoretical molecular weights calculated using massXpert [86] further confirmed the incorporation of AzK in Stx1B K9AzK and scFv OKT3 E129AzK (Additional file 1: Fig. S2, Table S1). Stx1B K9AzK monomers in solution oligomerized into a pentamer (Additional file 1: Fig. S1c), which is a prerequisite for the biological function of Stx1B. Finally, ITC analysis confirmed that Stx1B and Stx1B K9AzK bound Gb3 with millimolar K_D_ values (Additional file 1: Fig. S3) comparable to previous studies [87].

**Fig. 3:**
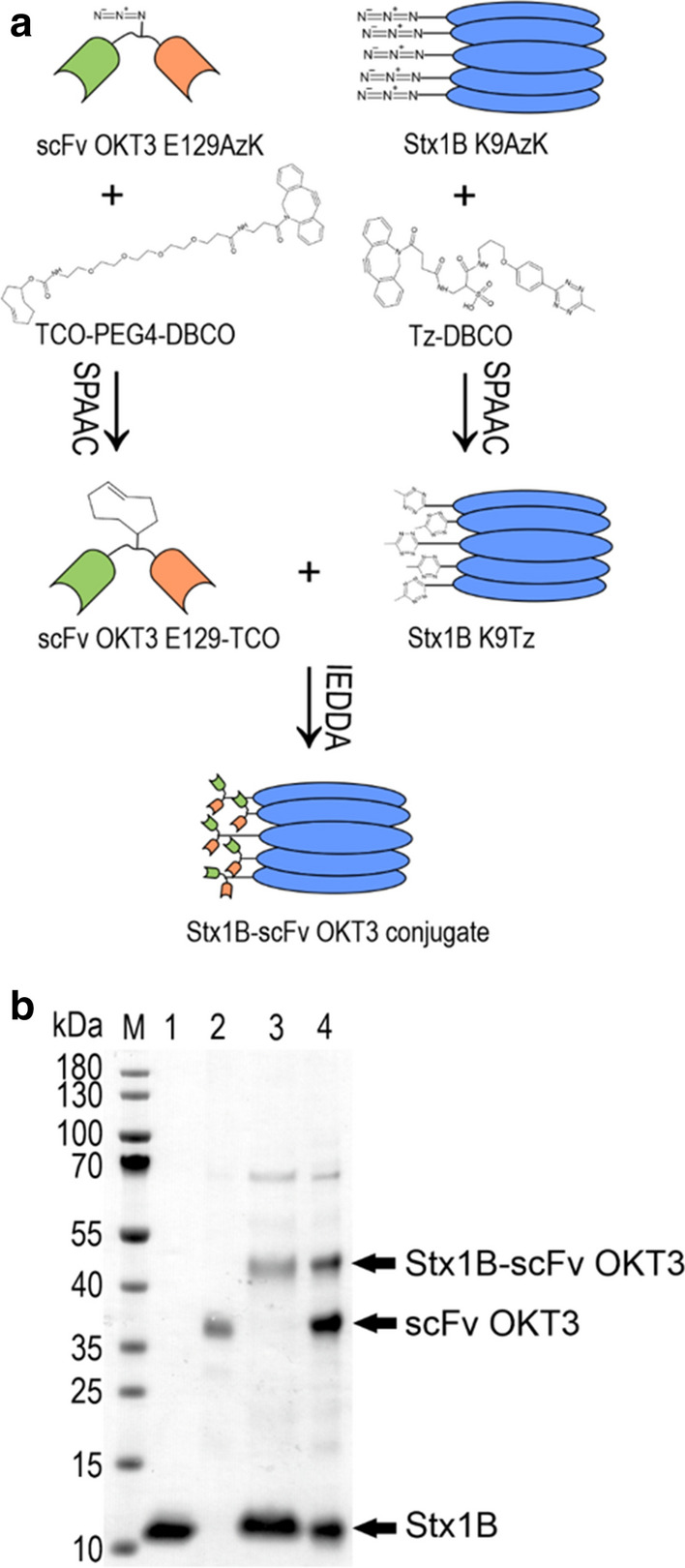
Formation of the Stx1B-scFv OKT3 conjugate. **a** Schematic representation of the conjugation approach. AzK was site-specifically incorporated at position E129 in scFv OKT3 and at K9 in Stx1B. Then, the azido groups in purified Stx1B K9AzK and scFv OKT3 E129AzK selectively reacted with the DBCO group on the methyltetrazine-DBCO and *trans*-cyclooctene-PEG4-DBCO linkers by SPAAC. When mixed, the methyltetrazine-functionalized Stx1B K9Tz and *trans*-cyclooctene-functionalized scFv OKT3 E129TCO coupled with each other via an IEDDA reaction to form the Stx1B-scFv OKT3 conjugate. **b** Qualitative SDS-PAGE analysis of the IEDDA reaction mixture (lane 4). Lane 1, Stx1B K9AzK; lane 2, scFv OKT3E129AzK; lane 3, SEC purified Stx1B-scFv OKT3 conjugate. Calculated molecular weights (MW_calc_) of the individual proteins are indicated in the legend to Fig. 2. M, molecular weight marker, the band sizes are indicated on the left margin of the gel. The gel was stained with Coomassie protein stain

The Stx1B-scFv OKT3 lectibody was assembled via two click reactions, SPAAC and IEDDA. The SPAAC reaction is a cycloaddition of a cyclic alkyne (e.g., dibenzocyclooctyne DBCO) and an organic azide (e.g., AzK) leading to a stable triazole product [88]. Whereas IEDDA involves the reaction between electron-poor dienes (e.g., tetrazines) and electron-rich dienophiles (e.g., *trans*-cyclooctenes) to form dihydropyridazines [89]. As illustrated in the scheme in Fig. 3a, we first exploited the azido groups on Stx1B K9AzK and scFv OKT3 E129AzK to install a DBCO-methyltetrazine- and a DBCO-PEG4-*trans*-cyclooctene linker, respectively, by SPAAC. In a second step, the tetrazine-functionalized Stx1B was conjugated with scFv OKT3 carrying the *trans*-cyclooctene-moiety by the IEDDA reaction to form the Stx1B-scFv OKT3 conjugate. The SDS-PAGE analysis of the IEDDA reaction mixture (lane 4 of the SDS gel shown in Fig. 3b) revealed the monomeric Stx1B-scFv OKT3 conjugate at an apparent molecular weight of 50 kDa (MW_calc_ 39 kDa) as well as unreacted Stx1B K9AzK at ~ 12 kDa (MW_calc_ 9 kDa) and scFv OKT3 E129AzK at 37 kDa (MW_calc_ 30 kDa). The band below 70 kDa most probably corresponds to an impurity in the scFv OKT3 E129AzK preparation. The in-gel tryptic digest of the band at 50 kDa excised from the SDS gel (lane 4, Fig. 3b) was confirmed as Stx1B-scFv OKT3 conjugate by mass analysis (Additional file 1: Table S2). The Stx1B-scFv OKT3 conjugate was purified from the reaction mixture by size exclusion chromatography (SEC) and re-run on an SDS gel, which showed the monomeric Stx1B-scFv OKT3 conjugate and unreacted Stx1B K9AzK but no unreacted scFv OKT3 E129AzK (Fig. 3b, lane 3). This finding indicates that not all five subunits in a Stx1B pentamer were conjugated with scFv OKT3. We deduced from the SEC analysis (Additional file 1: Fig. S4) that three scFv OKT3 molecules were conjugated to one Stx1B pentamer (Additional file 1: Table S3).

### Binding specificity and affinity of Stx1B-scFv OKT3 lectibody to tumor and effector cells

The ability of the generated lectibody to recognize the Gb3 and CD3 antigens as cell surface receptors was studied by flow cytometry. The glycosphingolipid Gb3 is commonly indicated as the B-cell differentiation antigen CD77 or BLA (Burkitt’s lymphoma-associated antigen). It is expressed on a subset of germinal center B cells [90] and present in high amounts on Burkitt’s lymphoma (BL) cells [91]. This prompted us to first investigate the anti-tumor activity of the Stx1B-scFv OKT3 lectibody in Burkitt's lymphoma-derived cell lines.

**Fig. 4:**
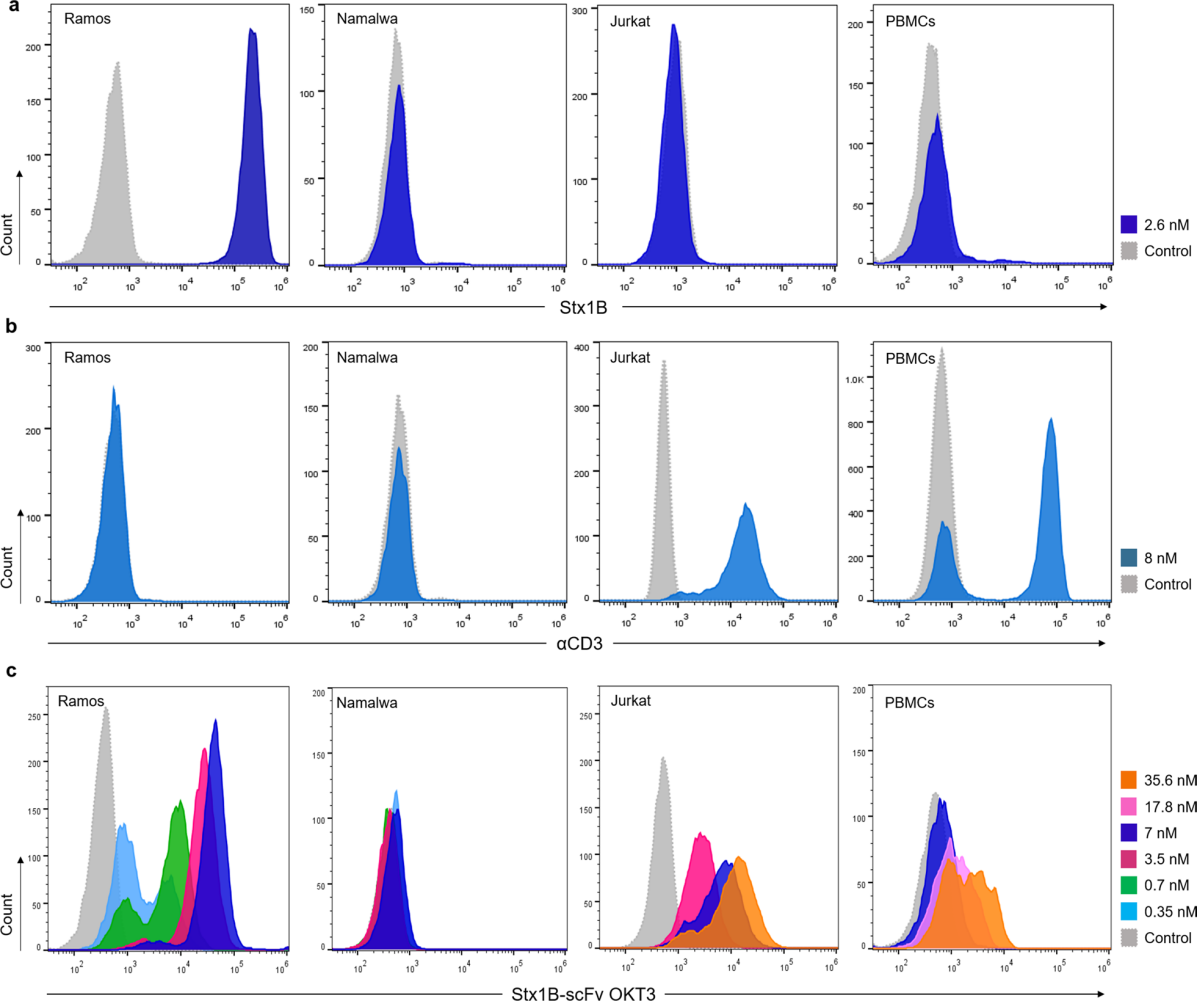
Binding of Stx1B, anti-human CD3 antibody and Stx1B-scFv OKT3 lectibody to tumor and effector cells. **a** Representative histograms of flow cytometry analysis of gated living Ramos, Namalwa, Jurkat T cells and PBMCs from healthy donors incubated with 2.6 nM Stx1B-Cy5 for the evaluation of Gb3 abundance at the plasma membrane. **b** Representative histograms of flow cytometry analysis of gated living Ramos, Namalwa, Jurkat T cells and PBMCs incubated with 8 nM anti-human CD3 Alexa-Fluor® 647 antibody and anti-human CD3 FITC antibody (αCD3). **c** Histograms of fluorescence intensity of gated living Ramos, Namalwa, Jurkat T cells and PBMCs incubated with increasing concentrations of Stx1B-scFv OKT3, detected by a fluorescent anti-6-His epitope tag AF647 antibody (dotted, grey: negative control; light blue: 0.35 nM; green: 0.7 nM; magenta: 3.5 nM; blue: 7 nM, pink: 17.8 nM; orange: 35.6 nM). Histograms show a dose-dependent trend in protein binding to the Gb3 antigen exposed at the surface of Ramos cells. Fluorescence intensity did not change following incubation of the lectibody with Gb3^−^ Namalwa cells, which excluded unspecific binding of the conjugate to the cell surface. Binding of the lectibody to CD3 receptors was proven by dose-dependent shift in fluorescence intensity for the tested Jurkat T cells and PBMCs. The number of cells within the live population (y-axis) is plotted against the fluorescence intensity of **a** Stx1B-Cy5, **b** αCD3, **c** Stx1B-scFv OKT3 (x-axis)

We included the Gb3-expressing (Gb3^+^) B cell line Ramos in our study, as well as the Gb3-negative (Gb3^−^) B cell line Namalwa as a negative control. In order to characterize the Gb3 aboundance on these cells, fluorescently labelled Stx1B-Cy5 was used in flow cytometry assays (Fig. 4a). To rule out the presence of CD3 antigens, cells were screened with an anti-human CD3 Alexa-Fluor^®^ 647 antibody (Fig. 4b). The analysis revealed high expression of Gb3 in Ramos cells upon incubation with 2.6 nM Stx1B, and confirmed the absence of this GSL on Namalwa cells. Both cell lines resulted negative for CD3 expression at their surface. Moreover, the specificity of the Stx1B K9AzK mutant towards Gb3 antigens on Ramos and Namalwa cells was then compared to that of Stx1B wild-type in flow cytometry analysis (Additional file 1: Fig. S5a, b), confirming the receptor specificity of the Stx1B K9AzK mutant.

Similarly, cultured human Jurkat T cells and peripheral blood mononuclear cells (PBMCs) isolated from healthy anonymous donors were initially characterized for the presence of Gb3 and CD3 antigens at their surface. The absence of Gb3 in these cells was monitored by incubation with 2.6 nM Stx1B-Cy5, in order to exclude unwanted cross-binding by the lectibody. The expression of CD3 receptors at the surface was recorded by incubation with an anti-human CD3 FITC antibody (Fig. 4a and b, respectively). Effector cells were confirmed to be CD3-positive (CD3^+^) and Gb3-negative (Gb3^−^). Additionally, scFv OKT3 wild-type and the scFv OKT3 E129AzK variant generated in this study were examined for their binding to Jurkat T cells and their identity was confirmed.

As a next step, we examined the binding of conjugated Stx1B-scFv OKT3 to tumor and effector cells. Thus, the lectibody was incubated with Ramos, Namalwa, Jurkat cells and PBMCs for 30 min on ice (Fig. 4c). A panel of different lectibody concentrations was used, spanning from 350 pM to 35.6 nM. At the end of incubation, cells were washed twice to remove the unbound protein and subsequently stained with fluorescently labeled anti-6-His epitope tag AF647 antibody to detect the presence of the Stx1B-scFv OKT3 lectibody at the cell surface in flow cytometry analysis. As depicted in Fig. 4c, Ramos cells, which are Gb3^+^, were positive with all the tested concentrations, showing a dose-dependent trend in lectibody binding. We did not record changes in fluorescence intensity for the Gb3^−^ Namalwa cells, indicating the absence of unspecific recognition and off-target interactions by the Stx1B-scFv OKT3 lectibody (Fig. 4c). Jurkat T cells and PBMCs were positive with all tested concentrations as well, and showed binding of the lectibody to CD3 receptors at the plasma membrane with higher concentrations of Stx1B-scFv OKT3 compared to Ramos cells, and in a dose-dependent manner.

**Fig. 5:**
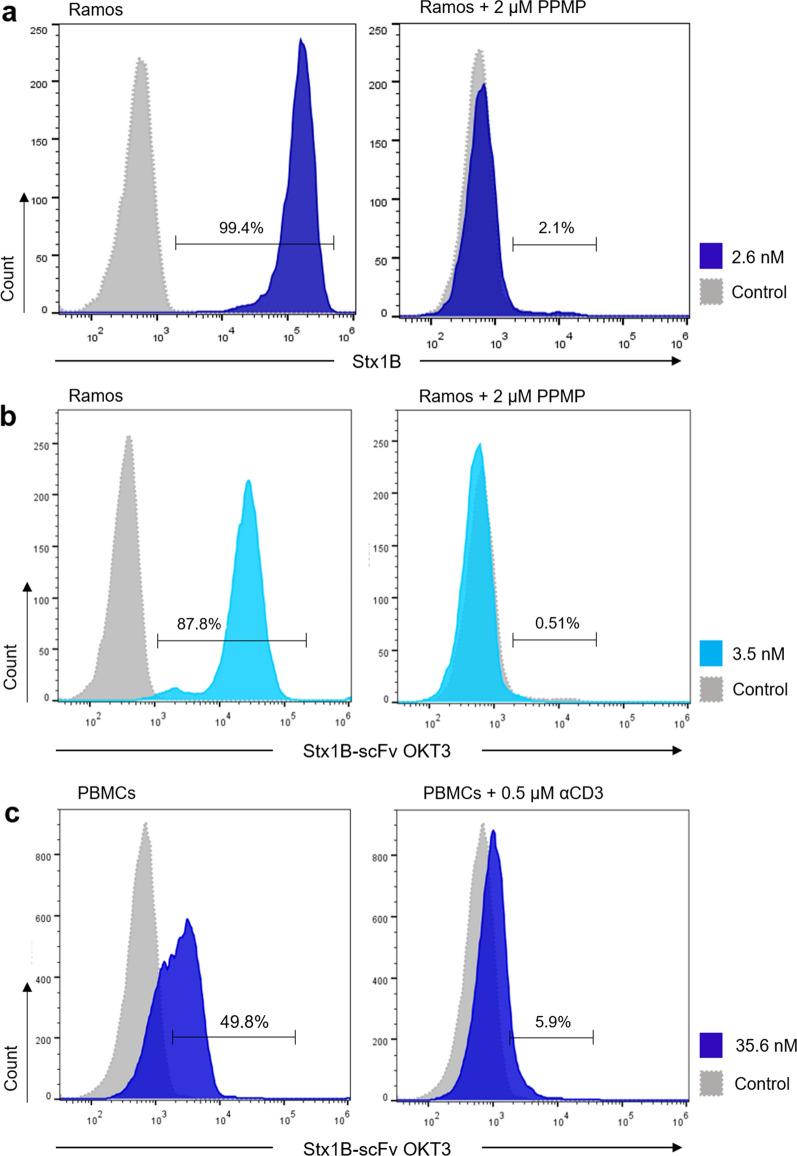
Binding of the Stx1B-scFv OKT3 lectibody to tumor and effector cells after depletion or saturation of receptors at the plasma membrane. Representative histograms of flow cytometry analysis of gated living **a**, **b** Ramos cells, and **c** PBMCs from healthy donors incubated with Stx1B or Stx1B-scFv OKT3 for 30 min on ice without (left panel) or after (right panel) depletion of receptors from the cell surface. **a** Histograms of fluorescence intensity of Gb3^+^ Ramos cells incubated with 2.6 nM Stx1B-Cy5 in the absence (untreated, left plot) or presence (right panel) of the GLS synthesis inhibitor PPMP. At 72 h post-treatment with 2 µM PPMP, Stx1B no longer bound to Ramos cells confirming depletion of Gb3 from the cell surface. **b** Histograms of fluorescence intensity of Gb3^+^ Ramos cells incubated with 3.5 nM Stx1B-scFv OKT3. Histograms on the left panel show binding of the lectibody to Gb3 exposed at the surface. In the right plot, flow cytometry analysis of Ramos cells pre-treated for 72 h with PPMP and incubated with Stx1B-scFv OKT3 is presented. In the absence of Gb3, the binding of the lectibody to the plasma membrane was drastically reduced. **c** Histograms of fluorescence intensity of PBMCs incubated with 35.6 nM Stx1B-scFv OKT3. On the left plot, histograms display lectibody binding to CD3 receptors present at the cell surface. The right plot shows flow cytometry analysis of PBMCs pre-treated with 0.5 µM of anti-human CD3 antibody (αCD3) for 20 min at room temperature, followed by incubation with 35.6 nM Stx1B-scFv OKT3. When CD3 receptors were occupied, binding of the Stx1B-scFv OKT3 lectibody to effector cells decreased remarkably. The number of cells within the live population (y-axis) is plotted against the fluorescence intensity of **a** Stx1B or **b** Stx1B-scFv OKT3 (x-axis)

To further demonstrate the specificity of the Stx1B-scFv OKT3 lectibody in targeting Gb3, Gb3^+^ Ramos cells were treated with the endogenous glucosylceramide synthase (GCS) inhibitor DL-threo-1-phenyl-2-palmitoylamino-3-morpholino-1-propanol (PPMP). To inhibit the synthesis of glucosylceramide-based GSLs and thus deplete Gb3 abundance at the plasma membrane, Ramos cells were incubated with 2 µM PPMP for 72 h before flow cytometry analysis. Cells were then stained with 2.6 nM Stx1B-Cy5 and analyzed in flow cytometry to confirm the depletion of Gb3 from the plasma membrane (Fig. 5a). Subsequently, cells were treated with 3.5 nM of Stx1B-scFv OKT3 for 30 min on ice, and samples were analyzed for the binding of the lectibody to target cells after staining with anti-6-His epitope tag AF647 antibody, as indicated above (Fig. 5b). Control Ramos cells, in which Gb3 synthesis was preserved, are shown in the left histogram of Fig. 5b, while PPMP-treated Ramos cells are reported on the right plot. Histograms of fluorescence intensities revealed a significant reduction in binding of the lectibody to the plasma membrane—from 87.8 to 0.51%—compared to untreated cells for the tested concentration (3.5 nM). These results corroborate those observed for Gb3^−^ Namalwa cells in Fig. 4a, confirming the exquisite specificity of the Stx1B-scFv OKT3 lectibody towards the Gb3 antigen.

In a similar manner, PBMCs were treated with an excess of anti-human CD3 antibody, competing with the lectibody for the CD3 receptors at the cell surface. Figure 5c displays flow cytometry analysis of control PBMCs (left plot) and anti-human CD3-treated PBMCs (right plot) after incubation with the Stx1B-scFv OKT3 lectibody. Upon saturation of CD3 receptors with 0.5 µM anti-human CD3 antibody, the fluorescence signal for the binding of the lectibody to PBMCs decreased drastically, from 49.8% in the untreated control to 5.9% for anti CD3-treated cells, as shown by the shift of the histogram towards lower values of fluorescence intensity. Taken together, these results support the functional affinity and specificity of the clicked scFv OKT3 and Stx1B domains of the lectibody for target and effector cells.

### Redirected killing of Gb3-positive Burkitt’s lymphoma-derived cells by the Stx1B-scFv OKT3 lectibody

**Fig. 6:**
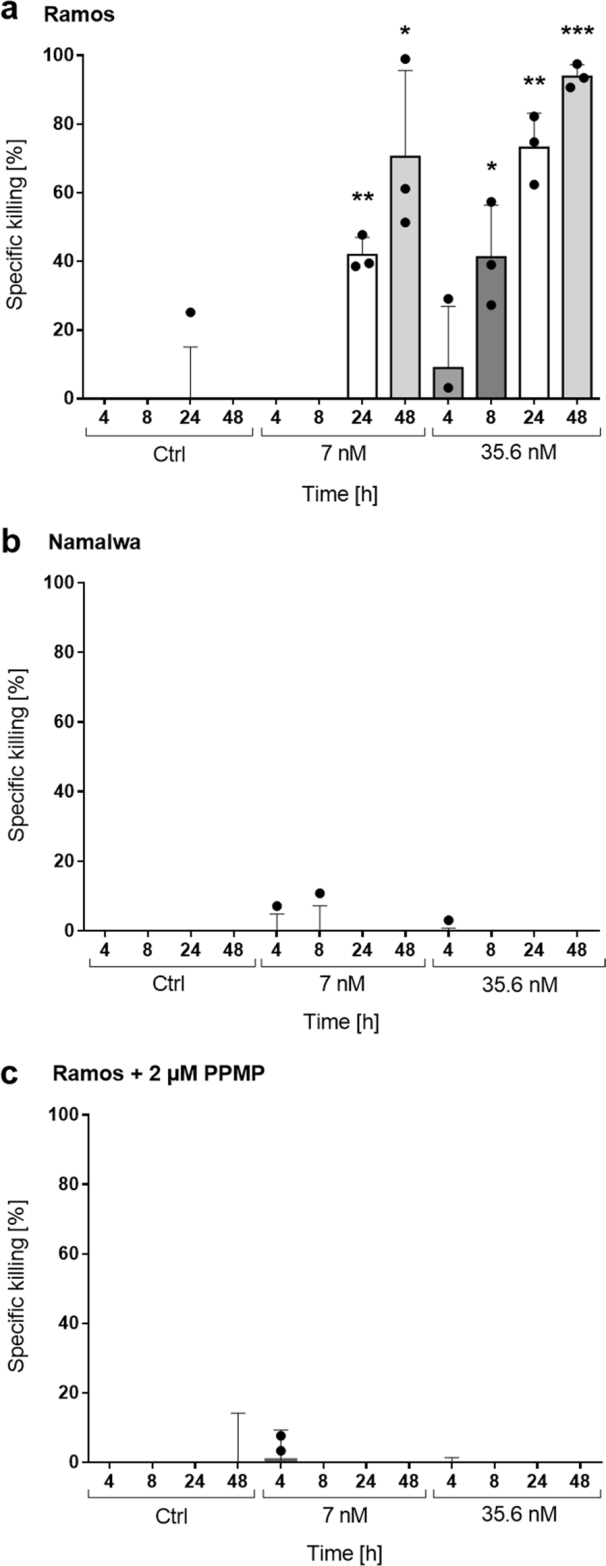
Induction of T cell-mediated cytotoxicity by Stx1B-scFv OKT3. Quantification of specific killing activity upon incubation of effector and target cells in the presence of 7 nM or 35.6 nM lectibody. **a** Gb3^+^ Ramos or **b** Gb3^−^ Namalwa cells were co-cultured with PBMCs in the effector-to-target (E:T) ratio of 5:1 and purified Stx1B-scFv OKT3 lectibody. After 48 h, about 93% of Gb3-expressing tumor cells were eliminated in presence of 35.6 nM, while Gb3^−^ cells did not show cytotoxicity. **c** T-cell specific killing was no longer detected when Ramos cells were pre-treated for 72 h with the GSL synthesis inhibitor PPMP. Upon depletion of Gb3 from the cell surface, the lectibody did not induce redirected lysis of tumor cells. Percent viability was calculated relative to the luminescence from an equal number of input control cells and used to calculate percent specific lysis. Results are expressed as a mean ± SD (*n* = 3) from 3 separate experiments. The experiments were performed with PBMCs derived from at least 3 different donors. Each dot represents data from individual donors. Data of cell proliferation are not shown in the graph. Statistical differences in independent samples were determined with a two-tailed, unpaired t-test for control and treatment groups, at each time point. Tests with a *p*-value ≤ 0.05 are considered statistically significant and marked with an asterisk (*). *p*-values ≤ 0.01 are shown as two asterisks (**), and ≤ 0.001 are summarized with three asterisks (***)

As proof-of-concept, we tested the ability of the generated lectibody to induce T cell-mediated killing of Gb3^+^ Burkitt’s lymphoma-derived tumor cells. The Stx1B-scFv OKT3 lectibody could redirect unstimulated peripheral T cells to lyse Gb3^+^ Ramos cancer cells in a concentration-dependent fashion. PBMCs and target cells were co-cultured in presence of the lectibody for 48 h, and elimination of target cells was recorded as bioluminescence (BLI) at several times (4, 8, 24 and 48 h). Efficacy of tumor cell lysis was investigated in presence of 7 nM or 35.6 nM lectibody, with the effector to target (E:T) ratio adjusted to 5:1. Control samples included PBMCs and target cells co-incubated in absence of lectibody, counted as spontaneous cell death. Figure 6 presents graphs of in vitro killing activity, expressed as percentage of specific killing induced by Stx1B-scFv OKT3. Strikingly, Gb3^+^ Ramos cells were efficiently eliminated by the treatment at low nanomolar concentrations. As reported in Fig. 6a, lower concentrations of Stx1B-scFv OKT3 (7 nM) induced around 40% of tumor cell lysis at 24 h, followed by an increase in tumor cell death to about 70% at 48 h. Remarkably, the higher lectibody concentration of 35.6 nM mediated tumor cell killing at earlier time points (around 40% at 8 h post-treatment) and affected 72% of tumor cell death after 24 h. At 48 h post-treatment with the lectibody we recorded 93% of Ramos cell elimination. Furthermore, flow cytometry analysis of Ramos cells and PBMCs revealed that the Stx1B-scFv OKT3 lectibody can be detected at the plasma membrane of treated cells after 24 and 48 h incubation, indicating a stable binding of the lectibody to surface antigens (Additional file 1: Fig. S6a, b). On the other hand, Gb3^−^ Namalwa cells were resistant to being targeted by the treatment for both tested concentrations, due to the absence of the Gb3 antigen at their surface (Fig. 6b). Similarly, cytotoxicity towards PPMP-treated Ramos cells (Fig. 6c) was not recorded, confirming poor or no activity of the Stx1B-scFv OKT3 in absence of the target receptor, the GSL Gb3.

### Induction of T cell activation by Stx1B-scFv OKT3

T cell activation is a tightly regulated cascade of events that lead to the induction of cytokines and expression of activation molecules, resulting in divergent immune responses [92]. Analysis of qualitative and quantitative T cell activation following immunological treatments provides valuable information about the type of immune responses mediated by such therapies. The existence of a tumor-specific CTL response in this study is strengthened by identification of T cell activation markers on the surface of CD8^+^ T lymphocytes. Upon T cell stimulation, the upregulation of several receptors is different for each stage of the activation process. Such activation markers can be detected on T cells’ surface using flow cytometry. Hence, we investigated the ability of Stx1B-scFv OKT3 lectibody to trigger effective signaling downstream of CD3 stimulation and induce the surface expression of CD69 (very early), CD71 (middle) and CD25 (late) activation markers on CD8^+^ cytotoxic T cells.

**Fig. 7:**
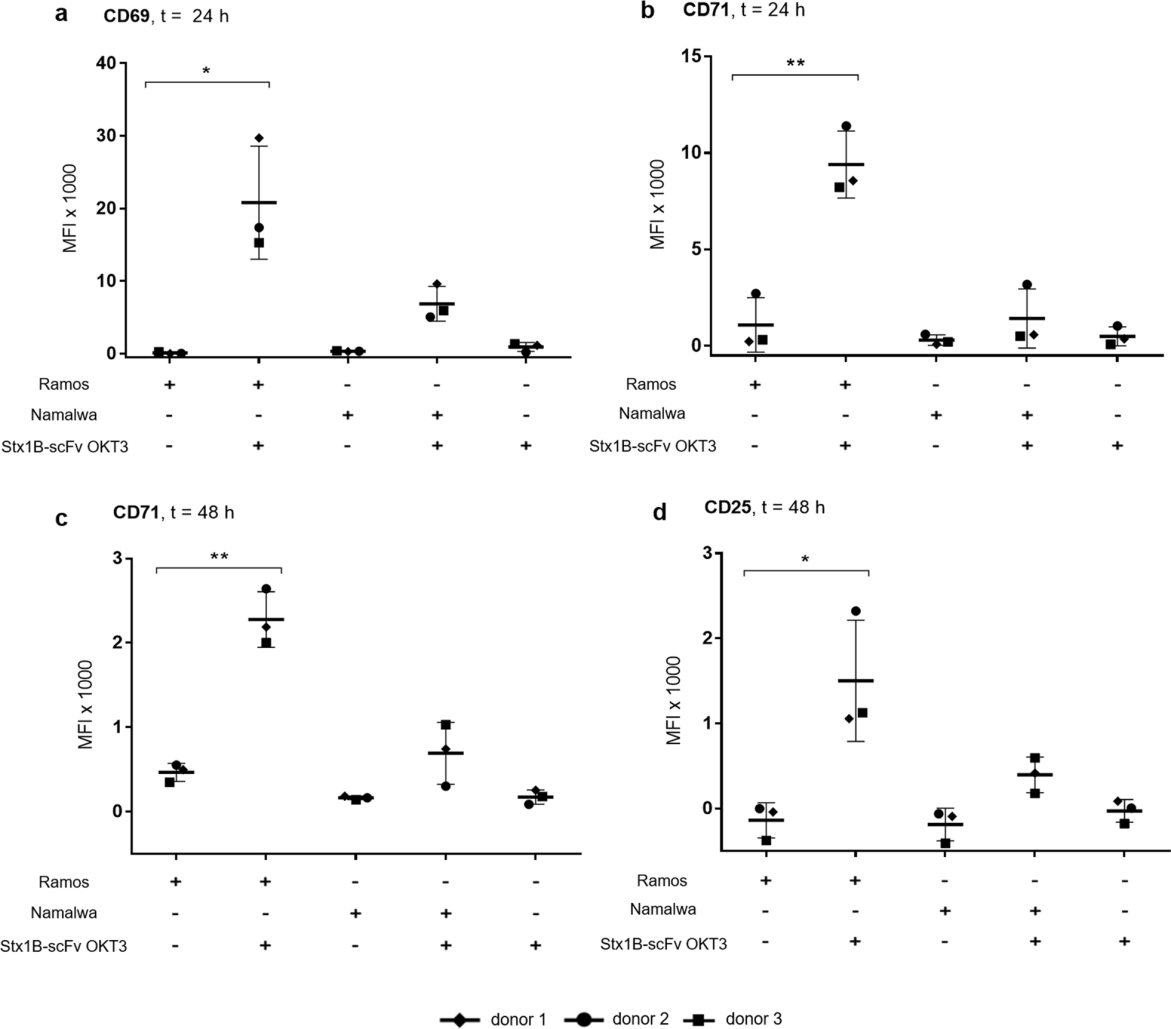
Up-regulation of CD8^+^ T cells surface markers CD69 (early), CD71 (middle) and CD25 (late) by Stx1B-scFv OKT3 lectibody. Gb3^+^ Ramos or Gb3^−^ Namalwa cells were co-cultured with PBMCs in the effector-to-target (E:T) ratio of 5:1 and purified Stx1B-scFv OKT3 lectibody (35.6 nM). Mean Fluorescence Intensity (MFI) data from three different donors were analyzed in flow cytometry from live CD8^+^ gated PBMCs. Results are expressed as a mean ± SD (*n* = 3) from separate experiments. After 24 h, the surface expression of T cell activation surface markers **a** CD69 and **b** CD71 was determined. **c** CD71 and **d** CD25 surface expression was monitored at 48 h following co-incubation of target and effector cells in presence of lectibody. CD8^+^ T cells were significantly activated when co-incubated with Gb3-expressing tumor cells and Stx1B-scFv OKT3, while they remained in a resting state when treated with Gb3-negative cells and lectibody. Statistical differences were determined with a two-tailed, unpaired t-test between control and each treatment group. Tests with a *p*-value ≤ 0.05 are considered statistically significant and marked with an asterisk (*), and *p*-values ≤ 0.01 are shown as two asterisks (**)

The earliest activation marker is CD69, an inducible cell surface glycoprotein expressed following activation via the TCR and its associated CD3 complex. CD69 is reported to play a role in the proliferation and survival of activated T lymphocytes [93, 94]. It is expressed at low basal levels in resting lymphocytes, while its transcription increases upon activation of T cells in a time-dependent manner between 3 and 12 h, and its expression remains elevated until 24 h, declining thereafter [95]. Accordingly, we detected the presence of elevated CD69 expression at the surface of CD8^+^ T cells following treatment with the lectibody. Unstimulated PBMCs from healthy donors were co-cultured with target cells in the presence of 35.6 nM lectibody for 24 or 48 h, and Mean Fluorescence Intensity (MFI) was calculated following flow cytometry analysis. At 24 h, CD69 results increased in the co-presence of Gb3^+^ Ramos cells and Stx1B-scFv OKT3, while its expression remained at basal levels when PBMCs were incubated solely with Stx1B-scFv OKT3, or increased only slightly when in the presence of Gb3^−^ Namalwa cells and Stx1B-scFv OKT3 (Fig. 7a).

The second activation marker of interest is represented by CD71, also known as Transferrin Receptor (TfR). CD71 is a cell surface iron transport receptor that is upregulated in T cells within 24–48 h following T cell activation. It is an essential factor for proliferating T cells [96] and can be considered as a mid-activation marker. Our results in Fig. 7b and c indicate an increase in the surface expression of this marker, similarly to our observation with CD69. CD8^+^ T cells presented the highest CD71 expression at both 24 and 48 h in the presence of Gb3-expressing tumor cells and lectibody.

The third receptor we investigated following T cell stimulation with Stx1B-scFv OKT3 is CD25, considered to be the most pronounced cellular activation marker. CD25 is the α-chain of the trimeric interleukin-2 (IL-2) receptor. It is constitutively expressed on the surface of several subsets of T lymphocytes, including regulatory and resting memory T cells. Following stimulation of the TCR/CD3 complex, CD25 is upregulated within 24 h and remains elevated for a few days [95, 97]. It is involved in T cell responsiveness to IL-2, resulting in lymphocyte activation and further IL-2 production, an event that determines survival, expansion and function of T lymphocytes. We determined the expression of CD25 on CTLs at 48 h post-treatment with the lectibody. As depicted in Fig. 7d, surface expression of CD25 in CD8^+^ T cells followed a similar trend to the previously described CD69 and CD71 activation markers. The induction of CD25 expression on T cells was relevant when they were stimulated with Stx1B-scFv OKT3, but only in the presence of Gb3^+^ tumor cells.

Collectively, our data show that the expression of CD69, CD71 and CD25 activation markers follows a precise and consistent time-course, where changes in CD71 expression proved to be the most prominent. Moreover, the recorded activation of CTLs is specifically driven by the treatment with the Stx1B-scFv OKT3, only for the Gb3^+^ target cells.

### Efficient recognition and T cell-mediated lysis of solid tumor cell lines induced by Stx1B-scFv OKT3

The approval of cancer immunotherapy for hematological malignancies has generated significant excitement, as it demonstrated remarkable efficacy in treating patients [98]. However, clinical success with immunotherapy for solid tumors has been mostly difficult to achieve, due to several limitations in delivering treatments. Tumor-associated biological barriers, which include immunosuppressive tumor microenvironment (TME), inefficient trafficking, and heterogeneity of tumor antigens represent the major challenges to overcome [99, 100]. As Gb3 overexpression has been described in a variety of solid tumors, including breast [13, 14], ovarian [15], pancreatic [18, 19] and colorectal [16, 17] carcinomas, we hypothesized that the lectibody generated in this study could also be effective against human cell lines derived from colon cancer.

**Fig. 8:**
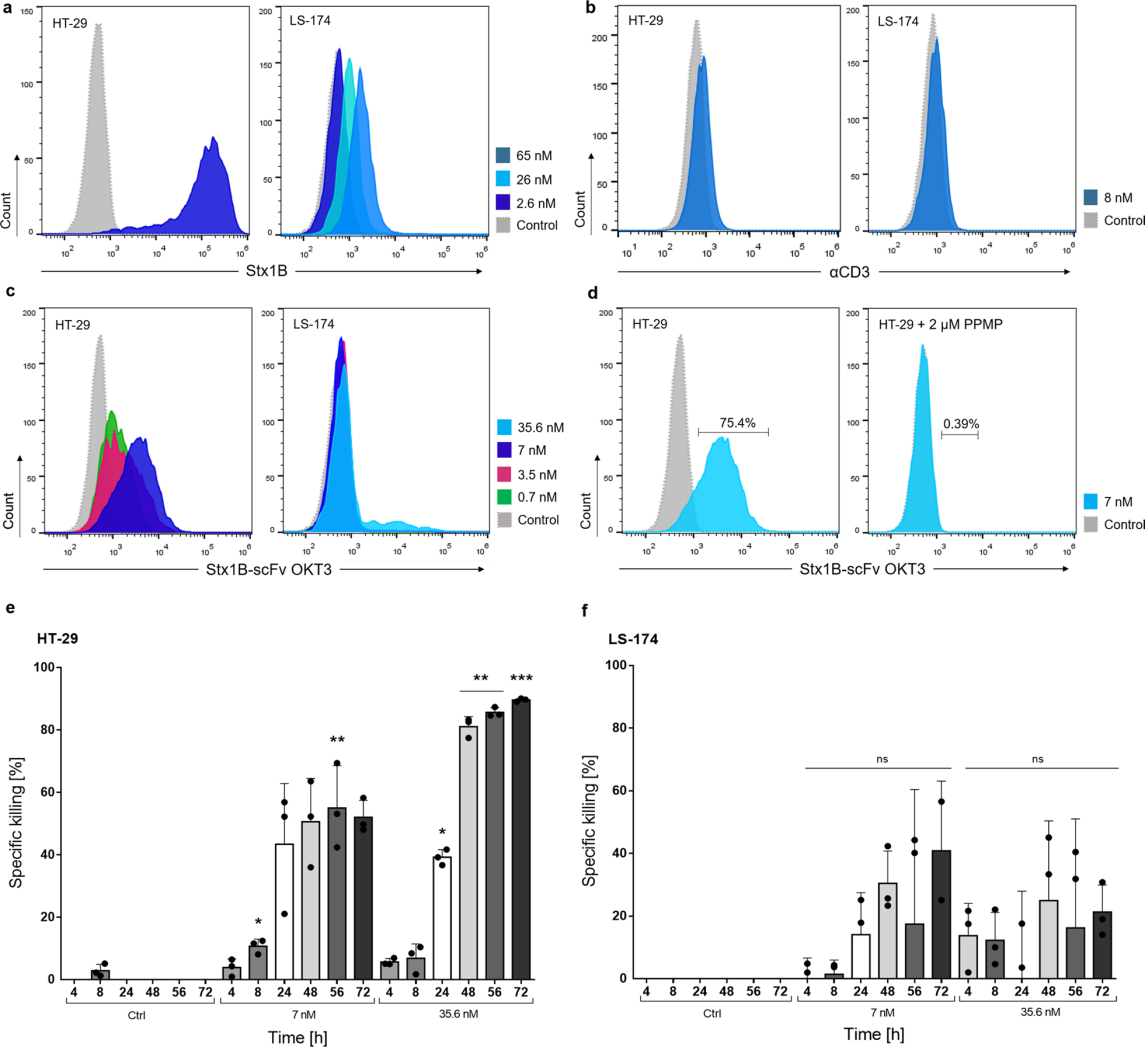
Recognition of solid tumor cells by Stx1B-scFv OKT3 and redirected in vitro tumor cell lysis.** a** Representative histograms of flow cytometry analysis of gated living HT-29 and LS-174 cells treated with 2.6, 26 or 65 nM Stx1B for the assessment of Gb3 abundance at the plasma membrane. HT-29 cells showed a high presence of Gb3, while LS-174 exhibited low amount of Gb3 at the cell surface. **b** Histograms of flow cytometry analysis of HT-29 and LS-174 cells incubated with 8 nM anti-human CD3 Alexa-Fluor^®^ 647 antibody (αCD3) confirming the absence of CD3 on tested cells. **c** Flow cytometry analysis of HT-29 and LS-174 treated with increasing lectibody concentrations (dotted, grey: negative control; green: 0.7 nM; magenta: 3.5 nM; blue: 7 nM, light blue: 35.6 nM), showing a dose-dependent trend in protein binding. **c** Flow cytometry analysis of HT-29 cells treated with Stx1B-scFv OKT3 without (left plot) or after (right plot) Gb3 depletion upon PPMP-treatment. When Gb3 synthesis was inhibited, binding of the lectibody to target cells decreased remarkably. The number of cells within the live population (y-axis) is plotted against the fluorescence intensity of **a** Stx1B, **b** αCD3, **c**, **d** Stx1B-scFv OKT3 (x-axis). **e** Quantification of specific cancer cell killing upon incubation of PBMCs and HT-29 cells or **f** PBMCs and LS-174 cells in presence of 7 nM or 35.6 nM lectibody for 72 h. Results are expressed as a mean ± SD (*n* = 3). The experiments were performed with PBMCs derived from 3 different donors. Each dot represents data from individual donors. Data of cell proliferation are not shown in the graph. Statistical differences in independent samples were determined with a two-tailed, unpaired t-test. Tests with a *p*-value ≤ 0.05 are marked with an asterisk (*). *p*-values ≤ 0.01 are shown as two asterisks (**), and ≤ 0.001 are summarized with three asterisks (***)

Firstly, we characterized two human colon adenocarcinoma cell lines—HT-29 and LS-174—for the abundance of Gb3 antigen at their plasma membrane by using fluorescently labelled Stx1B-Cy5 (Fig. 8a). By flow cytometry analysis, HT-29 were found to express a high amount of Gb3, as incubation with 2.6 nM Stx1B-Cy5 revealed a remarkable shift in fluorescence intensity. This result corroborates previously described studies which illustrated the abundance and variety of Gb3 isoforms present on HT-29 cells [101]. In contrast, the LS-174 cell line presented very low or no amounts of Gb3 [101], which we confirmed by our assay employing 2.6 nM Stx1B. Nevertheless, for higher concentrations of Stx1B (26 and 65 nM), a slight shift in fluorescence intensity was visible, indicating a basal presence of the Gb3 antigen at the surface of these cells. When treated with an anti-human CD3 Alexa-Fluor^®^ 647 antibody, both HT-29 and LS-174 resulted negative for the expression of CD3 receptors at their surface, which excludes accidental cross-reactivity of the lectibody (Fig. 8b). Additionally, a comparative analysis of the Stx1B wild-type and the Stx1B K9AzK mutant expressed in this study confirmed the specificity of the AzK mutant towards Gb3 on the surface of these tumor cells (Additional file 1: Fig. S7a, b).

Next, we evaluated the ability of the Stx1B-scFv OKT3 lectibody to recognize and bind to the Gb3 antigen on these cells. Figure 8c depicts histograms of fluorescence intensity of HT-29 and LS-174 cells incubated with a panel of different lectibody concentrations (700 pM–35.6 nM). Fluorescently labeled anti-6-His epitope tag AF647 antibody was used to assess the presence of the Stx1B-scFv OKT3 lectibody at the surface of treated cells. Flow cytometry analysis of HT-29, which are Gb3^+^ cells, revealed a dose-dependent trend in lectibody binding for the tested concentrations. We did not record drastic changes in fluorescence intensity for the LS-174 cells, although an underlying basal recognition of Gb3 can be appreciated at a lectibody concentration of 35.6 nM. This finding confirmed the Stx1B binding pattern highlighted in Fig. 8a. Additionally, Gb3^+^ HT-29 cells were treated with PPMP to inhibit the synthesis of the glucosylceramide-based GSLs, in order to deplete Gb3. The treatment was performed with the same experimental setup as reported for Ramos cells: HT-29 were treated with 2 µM PPMP inhibitor for 72 h before flow cytometry analysis. Fluorescently tagged Stx1B (2.6 nM) was used to confirm the absence of Gb3 at the plasma membrane (Additional file 1: Fig. S8). Similarly to the results observed for Burkitt´s lymphoma-derived cells, the Stx1B-scFv OKT3 lectibody could not recognize Gb3-depleted tumor cells (Fig. 8d). The binding of the lectibody to the PPMP-treated cells decreased drastically from from 75.4 to 0.34%, which validates the specificity of Stx1B-scFv OKT3 towards Gb3^+^ colon cancer cells.

We further aimed at investigating the efficacy of the lectibody in redirecting T-cell cytotoxicity towards solid cancer cell lines. To this end, PBMCs and target cells were co-cultured in presence of the lectibody for 72 h, and lysis of target cells was recorded as bioluminescence at several times (4, 8, 24, 48, 56 and 72 h). Tumor cell elimination was monitored upon treatment with 7 nM or 35.6 nM lectibody, as reported for Burkitt’s lymphoma-derived cell lines. The effector to target (E:T) ratio was maintained at 5:1. Control samples—PBMCs and target cells co-incubated in absence of lectibody—were used to determine spontaneous cell death. The percentage of specific T cell-mediated killing of HT-29 cells is shown in Fig. 8e. Tumor cell lysis increased according to incubation time and lectibody concentration. Remarkably, in the presence of 7 nM and 35.6 nM Stx1B-scFv OKT3, cytotoxicity of treated cells appeared as early as 4 h post-treatment, reaching ~ 40% of specific lysis at 24 h. While the treatment with a lower dose (7 nM) induced a maximum of 52% of tumor cell killing after 72 h, target cells treated with 35.6 nM Stx1B-scFv OKT3 displayed 81% of lysis at 48 h, culminating in 90% of tumor cell elimination at 72 h post-treatment. These results are in line with the obervations reported for Ramos cells in cytotoxicity assays, although with a slower kinetic in T cell-induced tumor killing. Accordingly, we tested LS-174 cells in combination with the Stx1B-scFv OKT3 and PBMCs, in the same experimental setup. Due to the basal presence of the Gb3 antigen at the surface of LS-174 cells—described by flow cytometry analysis by using higher marker concentrations—we recorded 10–40% of specific lysis between 24 and 72 h. The trend in killing activity seems to vary largely based on the specific activity of PBMCs isolated from different donors. Furthermore, the variation in tumor lysis does not follow a consistent tendency in time. Possibly, this finding highlights differences in Gb3 expression according to the stages of the LS-174 cell cycle and differentiation [102].

To summarize, these observations confirm that the lectibody engages cytotoxic T cells to redirect their killing activity towards Gb3-expressing colon cancer cells. The killing activity strongly correlates to the abundance of the Gb3 antigen at the surface of the target cells.

## Discussion

Selective targeting of cancer cells is a crucial requirement for the improvement of anti-tumor therapies to avoid toxicity in non-neoplastic cells [103]. It is becoming evident that certain altered glycans aiding tumor onset and progression can be used as selective targets for improved diagnostics and therapeutic strategies. Here, we present a novel bispecific lectibody that targets the glycosphingolipid Gb3 on Burkitt’s lymphoma-derived cells and colorectal adenocarcinoma cell lines by engaging T cells for a specific and powerful anti-tumor response. The format of a bispecific lectibody is inspired by the therapeutic class of BiTEs, which have proven efficient in redirecting immune cells, primarily T cells, towards target cells, thereby inducing anti-tumor activity. For example, the CD19 × CD3 canonical BiTE blinatumomab has achieved impressive efficacy in treating B cell malignancies [104]. Therefore, our study aimed to show that a bispecific lectibody with the lectin Stx1B as tumor-targeting domain and scFv OKT3 as a T cell engager could redirect T lymphocytes’ cytotoxicity towards Gb3-expressing tumor cells.

The potential of rationally engineered lectins to produce lectibodies has been already investigated in the past years [105]. Through the genetic fusion of a lectin and an antibody´s crystallizable fragment (Fc) of immunoglobulin G (IgG), the resulting lectibody molecules have shown potential as antiviral proteins. The recognition of carbohydrates on the envelope of viruses by the lectin domain, coupled to the antibody effector functions of the Fc fragment, have led to successful neutralization of viral entry into host cells and clearance of cells infected by viruses [106]. Recently, antibody-lectin chimeras (AbLecs) have been described as modular platform for glyco-immune checkpoint blockade in cancer immunotherapy, potentiating tumor phagocytosis and cytotoxicity in vitro [107].

Our new Stx1B-scFv OKT3 lectibody resembles the previously described lectibodies as it combines a lectin with an antibody fragment, nevertheless, the architecture is quite different. In lieu of the Fc fragment, we employed the scFv OKT3 as a T cell engager and selectively conjugated it to the Gb3-binding Stx1B lectin instead of genetically fusing it. This approach allowed us to select the attachment points for maximum functionality of both components and to join them with a predefined spacing using linker molecules. The lectin Stx1B was selected to successfully recognize Gb3-expressing tumors as previously described [18, 19, 44–46]. Due to the invariant property of CD3 chains in the TCR [108], CD3 was here chosen as a T cell surface target for the lectibody. The monoclonal antibody OKT3 recognizes a region of the antigenic CD3ε on human T cells and induces an immune response by T cell activation and proliferation [109]. In this study, we employed the OKT3 single chain variable fragment because it readily binds to the CD3 antigen, is stable and efficiently produced in *E. coli* [74, 110]. To crosslink Gb3-expressing tumor cells with T cells via CD3, the lectin Stx1B and the scFv OKT3 must be physically linked. The linkage can be affected at the gene level or by chemical ligation on the protein level. In general, genetic fusions open a narrow window for conjugating proteins either on the N-terminus or on the C-terminus. The approach leaves no room for site-selective conjugations, offers only limited options for linkers and poses limitations when the biological activity of one (or both) of the fusion partners depends on the free terminus [111]. Linkage at the protein level is often realized by chemical ligations involving lysine- and cysteine residues in proteins. Yet, more than one lysine or cysteine in the protein sequence will spoil the regioselectivity [112]. The conjugation reactions are inefficient if the lysine- or cysteine residues are buried in the protein structure [113]. A few chemically conjugated lectin-drug delivery vehicles were tested as potential biotherapeutics. For instance, wheat germ agglutinin (WGA)-conjugated liposomes loaded with amoxicillin showed potent antimicrobial activity [114]. To overcome the above-mentioned limitations of genetic fusions and bioconjugation at canonical amino acids such as lysine and cysteine, we embarked on a bioorthogonal conjugation strategy for Stx1B and the scFv OKT3. Bioorthogonal conjugation exploits unique reactive groups that are installed at predefined positions in the protein conjugation partners. To site-selectively install the unique reactivity, ncAAs carrying a corresponding reactive side chain can be introduced into the target protein(s) at an in-frame amber stop codon [112]. Here we used the pyrrolysyl-tRNA synthetase from *Methanosarcina mazei* and its cognate amber suppressor *Mm*tRNA_CUA_ for this purpose. The *Mm*PylRS/*Mm*tRNA_CUA_ pair is orthogonal in *E. coli*, which means that the *Mm*PylRS does not charge any of the *E. coli* tRNAs, nor is the *Mm*tRNA_CUA_ charged with a canonical amino acid by any of the host aminoacyl-tRNA synthetases. Wild-type *Mm*PylRS accepts a palette of (pyrol)lysine derivatives such as AzK [85]. We selected the sites for incorporation of AzK into Stx1B and scFv OKT3 such that the reactive azido-group would be surface exposed and as distant as possible from the glycan- or antigen-binding sites, respectively. These conditions were excellently fulfilled by residues K9 and E129 of Stx1B and scFv OKT3 (Fig. 1).

The direct bioorthogonal conjugation of two proteins requires that they are functionalized with compatible reactive groups. Unless an orthogonal pair accepts both corresponding ncAAs, individual orthogonal pairs are necessary [85]. Orthogonal pairs for the incorporation of azido-ncAAs [85, 115], cyclooctynyllysine derivatives [116], ncAAs with *trans*-cyclooctene- and bicyclooctyne- [117] as well as tetrazine- [118] side chain moieties were previously devised. Our approach relies on a two-step strategy (Fig. 3a): we first functionalized both proteins with an azido-group by the site-specific incorporation of AzK as outlined above. AzK was efficiently incorporated into scFv OKT3 and Stx1B as reflected by titers corresponding to 70% and 93% of the corresponding wild-type proteins. The azide groups allowed us to perform a SPAAC reaction with bi-functional linker molecules that carried each an azide-reactive DBCO group on the one end and a *trans*-cyclooctyne- or a tetrazine-moiety for IEDDA on the other. The resulting methyltetrazine functionalized Stx1B K9Tz and *trans*-cyclooctene functionalized scFv OKT3 E129TCO spontaneously assembled into the Stx1B-scFv OKT3 lectibody in the second conjugation step. Our two-step conjugation strategy allowed selection of the linker lengths and flexibility in the orientation of Stx1B and scFv OKT3 in the lectibody such that the bispecific properties were retained. This flexibility is particularly crucial for the conjugation of large molecules such as proteins. Our prediction of the lectibody structure suggested that this would be the case. Indeed, the flow cytometry analysis confirmed that the Stx1B and scFv OKT3 modules preserved their affinity and recognition towards Gb3 and CD3 antigens, respectively, upon conjugation. Moreover, we observed the selective killing of Gb3-expressing cancer cells, which indicates that the T cells were brought in close contact with the cancer cells.

While the structural prediction of the lectibody indicated that five scFv OKT3 molecules can be conjugated to a Stx1B pentamer, our SEC results showed that only three scFv OKT3 molecules were conjugated (Additional file 1: Table S3). This result adds another hint to the fact that bioorthogonal conjugation efficiencies rarely derive a 100% conjugate yield. Varying conjugation efficiencies were reported previously, even with small molecules such as fluorescein and with DNA oligomers. For instance, Synakewicz et al. reported 47% yield for conjugating proteins with azide-modified DNA oligomers by SPAAC [75], and Maggi et al. observed a remarkable 62% conjugation of trastuzumab-tetrazine with TCO-fluorescein by the IEDDA reaction [119]. These findings illustrate that conjugation efficiencies vary not only with the conjugation partners, i.e., protein with small molecule or DNA oligomer, but also with the conjugation method used. In this study, we were able to successfully conjugate three large ~ 30 kDa scFv OKT3 molecules to one ~ 45 kDa Stx1B pentamer via linkers by two successive SPAAC and IEDDA click reactions. It is key to understand what role the hydrophopathy of the linkers plays in the conjugation reaction to tackle the incomplete decoration of the Stx1B pentamer with the scFv OKT3 modules. For instance, Rahim et al. showed that 90% of the TCO groups without a linker attached on the surface of monoclonal antibodies were masked by their hydrophobic interactions with the antibodies. They had overcome this problem by adding hydrophilic PEG to the linker between the reactive groups [120]. In the similar way, to improve the conjugation efficiency of Stx1B with scFv OKT3 in the future, water-soluble methyltetrazine-PEG-DBCO linkers of short varying lengths might be used to reduce non-specific interactions.

As demonstrated, the Stx1B-scFv OKT3 promotes up to 93% of Burkitt’s lymphoma-derived tumor cells elimination within 48 h and 90% of solid tumor cells lysis at 72 h post-treatment. Low nanomolar concentrations (7 nM and 35.6 nM) of the lectibody induced a potent T cells response against Gb3^+^ Ramos and HT-29 cells, promoting CD8^+^ T cells activation. Noticeably, the Stx1B-scFv OKT3 lectibody did not affect the viability of Gb3^˗^ Namalwa cells, excluding the presence of undesired off-target cytotoxicity, while promoting a less pronounced cell lysis of LS-174 cells, characterized by a low expression of the Gb3 antigen, which was only detectable by using higher marker concentrations.To this matter, the excellent specificity of BiTEs has made this platform an exquisite system for treating cancer. Due to their bispecific configuration, the induction of a strong T cell response is accomplished by physically bridging cells, and the linkage of T cells to tumor cells is crucial to the BiTE´s cytotoxic mechanism. Single-sided binding of BiTE molecules is not sufficient in driving the activation of T cells [121], nor to induce cytokines expression—including interferon gamma, tumor necrosis factor alpha, IL-6, and IL-10. Lack of dual binding, as proven for Gb3^˗^ Namalwa cells, demonstrated the ability of Stx1B-scFv OKT3 to circumvent undesired T cell activation. This finding suggests the strict dependence of this lectibody on simultaneous T cell-tumor cell engagement, only in the presence of the GSL Gb3.

According to SEC analysis, the purified lectibody consisted of a conjugate of 140 kDa in size. This lectibody is roughly three times bigger in size than other recombinant bispecific antibodies able to redirect T lymphocytes to tumor cells. Generally, most bispecific antibody platforms—including BiTEs, tandem scFv molecules (taFv), diabodies (Db), or single chain diabodies (scDb) [122–124]—consist of small molecules with molecular masses of 50–60 kDa. However, due to their small size, these formats often suffer from poor pharmacokinetic (PK) and pharmacodynamic (PD) properties in vivo. For example, they are rapidly cleared from circulation and their half-life is less than 30 min, which makes it difficult to target them to the site-of-action for long duration times [125–127]. This trait hampers their therapeutic applications, as they require multiple doses and repeated injections or infusions of treatment. Several antibody-based platforms have been engineered to improve their PK properties, and most attempts have been directed so far to increase their molecular sizes. The addition of a third scFv to the polypeptide chain of bispecific antibodies led to the design of single chain triplebody formats (sctbs) with a mass of about 90 kDa, exhibiting one domain for the recruitment of effector cells and two specific binding sites for antigens on tumor cells [128]. Such design successfully prolonged the half-life of the format, whose molecular size was above the kidney exclusion limit. Other common strategies comprise chemical coupling of polyethylene glycol (PEG) chains [129], multimerization design [126, 130], or fusion with long-circulating serum proteins such as albumin [131, 132]. Due to its larger size, the Stx1B-scFv OKT3 lectibody presented in this study has a great potential to avoid clearance from serum within few hours. To this end, future in vivo studies are required to establish the clearance rate and renal excretion of Stx1B-scFv OKT3, assessing the lectibody’s circulation times and protection from catabolism. On the other end, potential off-target cytotoxicity should be evaluated as a consequence of the lectibody persistence and recirculation in plasma. This includes the induction of T cell-mediated lysis in cells and tissues which present only a mild expression of Gb3, and the overactivation of the immune system after a stable binding of the scFv OKT3 to CD3 receptors on the surface of CD4^+^ and CD8^+^ T cells.

The remarkable performance of the Stx1B-scFv OKT3 lectibody in enabling T cell-mediated lysis of tumor cells is accompanied by activation of the cytotoxic CD8^+^ T lymphocytes. The treatment elicited a notable upregulation of crucial markers at the surface of T cells—namely CD69, CD71, and CD25—at 24 and 48 h post-treatment. The appearance of such activation markers led to the identification of different stages in the T cells activation process, which was exclusively driven by the co-presence of lectibody and Gb3^+^ target cells. CD69, CD71 and CD25 were significantly increased and reached a peak in surface expression that overlaps with the onset of cytotoxic activity, precisely at 24 and 48 h. This increment in expression was elicited solely when Gb3^+^ Ramos cells and Stx1B-scFv OKT3 were added to the culture, indicating the simultaneous engagement of effector and target cells and the target-specific killing induced by our treatment. These findings are in line with the therapeutic mechanism of action reported for BiTEs, where significant cytotoxicity by T cells is registered even in the absence of co-stimulation [121, 133].

Bifunctional molecules represent a promising anti-cancer arsenal, targeting a variety of tumor-associated antigens on both solid and hematologic tumors [61]. In a combinatorial approach, our lectibody might improve the outcome of existing therapies for the eradication of those tumor cells which present dramatic alterations of glycosylation at the surface. When targeting cancer-associated glycans, glycan-binding proteins (GBPs), such as lectins and anti-glycan antibodies, can be used to discriminate between tumor and normal cells. As tumor-targeting ligands, lectins can be used to increase the selectivity and efficacy of anti-cancer treatments and enhance their concentration at the tumor sites. Interestingly, in addition to Stx1B, several other bacterial lectins have found application in tumor detection or treatment. For example, the cholera toxin (Ctx) from *Vibrio cholerae*, belonging to the AB_5_ family of microbial toxins, has been proven effective in a number of studies for tumor targeting and imaging [38]. More recently, we demonstrated the efficiency of the engineered FS-Janus lectin consisting of two carbohydrate-binding domains in detecting and targeting pathological hypersialylation on non-small cell lung cancer via its multivalent architecture, and with remarkable nanomolar avidity [134, 135]. The bispecific lectin was reported to crosslink glyco-decorated giant unilamellar vesicles and lung epithelial tumor cells, leading to the intracellular uptake of liposomal content and unraveling its potential in lectin-mediated drug delivery. Moreover, Meléndez et al. developed a panel of lectin-based chimeric antigen receptors (CARs) T cells, which demonstrated high therapeutical potential towards a variety of hematological malignancies and solid tumors expressing Gb3. In their studies, the Gb3-binding lectins StxB from *Shigella dysenteriae*, LecA from *Pseudomonas aeruginosa*, and the engineered Mitsuba from *Mytilus galloprovincialis* were employed to recognize the TACA Gb3 and fused to a second-generation CAR, achieving excellent target-specific cytotoxicity against Burkitt’s lymphoma-derived cell lines as well as colorectal and triple-negative breast-cancer [101]. Overall, these studies together with our current study support the potential of lectins as tools in many therapeutical applications where the glycome plays a crucial role in the development and sustainment of pathological conditions.

The described observations of dual-binding dependence, target-specific killing and absence of co-stimulation suggest a model for our lectibody-mediated cytotoxicity, where multiple lectibody-dependent binding events occur between T cells and tumor cells in culture, promoting clustering of T cell receptors and activation of cytotoxic signaling. Indeed, the presence of several scFvs OKT3 in the lectibody allows this bispecific T cell engager to be presented in a polyvalent form. This might induce the formation of an immunological synapse and release of cytolytic granules resulting in tumor cell lysis, as observed for BiTEs. There is much further knowledge to gain regarding the mechanism of action of the lectibody. Foremost, the investigation of its efficacy and potency in animal models must be carried out to further characterize the potential of this bispecific format in cancer therapy, as well as a better understanding of how the lectibody is processed by the body and of the side effects that might appear during therapy. In addition, examining Stx1B-scFv OKT3 in combination with other therapies will be necessary. For instance, it will be interesting to see how the lectibody performs in association with immune checkpoint inhibitors, which promote a greater T cell activation. A combinatorial strategy of this type could increase Stx1B-scFv OKT3 efficacy by enhancing and maintaining T cell activity for tumor eradication [136]. Nevertheless, it is important to point out that the GSL Gb3 is present on the cell membrane of a variety of non-transformed cells, which could lead to the onset of off-target cytotoxicity upon administration of the lectibody. A key factor that must be taken into account is the difference in Gb3 expression levels between pathological and physiological states, highlighted by the impact they have on StxB participation to receptor recognition [28, 137–139]. It has been proven that the binding of the toxin and its targeting to a specific intracellular transport pathway within Gb3^+^ cells is determined by the heterogeneity in Gb3 isoforms and the abundance of Gb3 in the lipid rafts of the plasma membrane [140]. It becomes evident the necessity for a screening of cytological specimens to determine the Gb3 status of patients prior to making such a therapeutic choice.

Finally, the lectibody concept described here possesses versatile features, since it can be adapted to other TACAs by exchanging Stx1B with another lectin. Recent advances in the use of lectins in research and medicine suggest they are potential tools for many applications, such as drug delivery and selective targeting of pathological conditions with a focus on glycosylation changes [47, 48]. Lectins engineering, as proven in this study, may offer the possibility to target glycan epitopes on tumor cells and boost the efficacy of current tumor therapies.

## Conclusions

To summarize, our findings show a prospective proof-of-concept for lectin-induced T cell cytotoxicity towards Gb3^+^ malignant cells. A prototype of a lectibody with bifunctional properties was produced using a click conjugation strategy and its functionality was evaluated in cell-binding and cytotoxicity assays. Although the mechanism of action of Stx1B-scFv OKT3 has to be further explored in vivo, we demonstrate encouraging properties of a bispecific lectin-based platform that selectively targets altered glycostructures on tumor cells. Additional improvements will focus on the evaluation of linkers with increased length and longer incubation times of click reactions to optimize product yield. Nonetheless, the lectibody format represents a promising tool for cancer therapy and could enhance selective targeting of the glycome in many human malignancies.

## Supplementary Information

**Additional file 1: Figure S1.** Expression, purification and characterization of Stx1B K9AzK. Coomassie stained SDS gels showed. **a** Expression of Stx1B K9AzK with the addition of AzK at a final concentration of 5 mM during induction with IPTG. **b** No expression of the target protein was detected when the culture medium was not supplemented with AzK during induction. Expression of Stx1B K9AzK with the addition of AzK at a final concentration of 5 mM during induction. M, molecular weight marker, the sizes of the marker bands are indicated; lane 1, clarified lysate; lane 2, flow through after loading clarified lysate on a zinc-charged sepharose column; lane 3, column wash; lanes 4 to 11, elution fractions. **c** 1.5 µg purified Stx1B K9AzK were subjected to native PAGE, a silver-stained 3–12% PA gel is shown. M, native PAGE marker, marker band sizes indicated in kDa; lane 1, Stx1B K9AzK (MW_calc_ ~ 45 kDa) in its native pentamer conformation. The black arrows indicate the protein bands of interest. **Figure S2.** Electrospray ionization mass spectrometry (ESI–MS) measurements of intact **a** Stx1B, **b** Stx1B K9AzK, **c** scFv OKT3 and **d** scFv OKT3 E129AzK. The proteins identified from the peaks are detailed in the Additional file 1: Table S1. **Figure S3.** Isothermal titration calorimetry (ITC) analysis of the interaction of **a** Stx1B and **b** Stx1B K9AzK with globotriaose. Raw data (top panel) and the binding isotherms obtained by plotting integrated data from titrations (bottom panel) are shown. **Figure S4.** Size analysis of the Stx1B-scFv OKT3 conjugate. **a** Representative size exclusion chromatogram of the IEDDA reaction mixture. **b **Protein standards aprotinin (Apr)- 6500 Da, ovalbumin (O)- 43000 Da, conalbumin (C)- 75000 Da, aldolase (Ald)- 158000 Da and ferritin (F)- 440000 Da were run on the S200 Increase 10/300 GL column for calibration. The column void volume (V_0_) 7.88 mL was determined by Blue dextran 2000. The gel-phase distribution coefficient (*K*_*av*_) was calculated using the formula *K*_*av*_ = *(V*_*e*_*-V*_*o*_*)/(V*_*b*_*-V*_*o*_*)* where *V*_*e*_ is elution volume and *V*_*b*_ is the column bed volume (24 mL). A calibration curve was plotted with *K*_*av*_ versus the logarithm of molecular weight. The calibration curve calculated from molecular weight standards is a straight line with a coefficient of determination (R^2^) of 0.9972. The red circle represents the Stx1B-scFv OKT3 conjugate and the green circle unreacted scFv OKT3 E129TCO. The straight-line equation K_av_ = (-0.3208) x (MW) + 1.9415 deduced from the calibration curve was used to determine the experimental molecular weights of the unknown as stated in Additional file 1: Table S3. **Figure S5.** Comparative analysis of Stx1B wild-type and mutant proteins binding to tumor cells. Representative histograms of flow cytometry analysis of gated living Ramos and Namalwa cells, incubated with **a** Stx1B wild-type or **b** Stx1B K9AzK for 30 min on ice. **a** Histograms of fluorescence intensity of Gb3^+^ Ramos and Gb3^−^ Namalwa cells incubated with Stx1B wt produced in this study (dotted, gray: negative control; light blue: 2.6 nM). **b** Histograms of fluorescence intensity of Ramos and Namalwa cells incubated with Stx1B K9AzK (dotted, gray: negative control; light blue: 2.6 nM; blue: 13 nM; dark blue: 26 nM). Stx1B K9AzK exhibited a similar binding pattern to Gb3 in comparison to the wild-type protein at the cell surface of Gb3^+^ Ramos cells. Fluorescence intensity did not change following incubation of AzK-incorporating Stx1B with Gb3^−^ cells, excluding unspecific binding of the protein to the cell surface, even at higher Stx1B K9AzK concentrations (13 nM and 26 nM). All treated cells were stained with anti-6-His epitope tag AF647 antibody to detect the presence of wild-type and mutant proteins at the plasma membrane. The number of cells within the live population (y-axis) is plotted against the fluorescence intensity of tested proteins (x-axis). **Figure S6.** Binding of the Stx1B-scFv OKT3 lectibody to tumor and effector cells after 24 and 48 h co-incubation. Representative histograms of flow cytometry analysis of gated living **a** Ramos, and **b** PBMCs from healthy donors co-incubated in presence of Stx1B-scFv OKT3 for 30 min on ice (t = 0 h) or for 24 and 48 h at 37 °C (t = 24 h, t = 48 h). **a** Histograms of fluorescence intensity of Gb3^+^ Ramos incubated with Stx1B-scFv OKT3 for different time points (dotted, gray: negative control; light blue: 3.5 nM). Histograms depict a stable lectibody binding to Ramos cells after 24 h (right plot). **b** Histograms of fluorescence intensity of PBMCs incubated with Stx1B-scFv OKT3 for different time points (dotted, gray: negative control; blue: 35.6 nM). Histograms display lectibody binding to CD3 receptors on treated cells. At t = 24 h and t = 48 h following incubation with Gb3^+^ Ramos and Stx1B-scFv OKT3, the lectibody could still be partly detected at the membrane of effector cells. The number of cells within the live population (y-axis) is plotted against the fluorescence intensity of Stx1B-scFv OKT3 (x-axis). **Figure S7.** Comparative analysis of Stx1B wild-type and mutant proteins binding to solid tumor cells. Representative histograms of flow cytometry analysis of gated living HT-29 and LS-174 cells, incubated with **a** Stx1B wild-type or **b** Stx1B K9AzK for 30 min on ice. **a** Histograms of fluorescence intensity of HT-29 and LS-174 cells incubated with Stx1B wt produced in this study (dotted, gray: negative control; light blue: 2.6 nM; blue: 26 nM, red: 65 nM). **b** Histograms of fluorescence intensity of HT-29 and LS-174 cells incubated with Stx1B K9AzK (dotted, gray: negative control; light blue: 2.6 nM; blue: 26 nM; red: 65 nM). Stx1B K9AzK exhibited a similar binding pattern to Gb3 in comparison to the wild-type protein at the cell surface of HT-29 and LS-174 cells. All treated cells were stained with anti-6-His epitope tag AF647 antibody to detect the presence of wild-type and mutant proteins at the plasma membrane. The number of cells within the live population (y-axis) is plotted against the fluorescence intensity of tested proteins (x-axis). **Figure S8.** Flow cytometry analysis of PPMP-treated HT-29 cells. Representative histograms of gated living HT-29 cells incubated with Stx1B-Cy5 for 30 min on ice without (left panel) or after (right panel) Gb3 depletion. At 72 h post-treatment with 2 µM PPMP, Stx1B no longer bound to HT-29 cells confirming Gb3 depletion from the cell surface. **Table S1**. Analysis of ESI–MS spectral data. **Table S2.** Stx1B-scFv OKT3 conjugate identified by mass analysis. **Table S3.** Experimental molecular weights calculated from the calibration curve shown in Additional file 1: Fig. S4.

## Data Availability

The datasets generated and/or analyzed during the current study are available from the corresponding authors on reasonable request.

## Abbreviations

ADCs: Antibody–drug conjugates
AzK: Azidolysine
BiTEs: Bispecific T cell engagers
BLA: Burkitt’s lymphoma-associated antigen
BLI: Bioluminescence
bsAbs: Bispecific antibodies
CTLs: Cytotoxic T lymphocytes
DBCO: Dibenzocyclooctyne
DMEM: Dulbecco's modified eagle medium
E:T: Effector to target
FACS: Fluorescence-activated cell sorting
Gb3: Globotriaosylceramide
GCS: Glucosylceramide synthase
GSL: Glycosphingolipid
IEDDA: Inverse electron demand Diels–Alder
mAb: Monoclonal antibody
MFI: Mean fluorescence intensity
ncAA: Non-canonical amino acid
PBMC: Peripheral blood mononuclear cell
PBS: Phosphate-buffered saline
PDB: Protein data bank
PEG: Polyethylene glycol
PPMP: 1-Phenyl-2-palmitoylamino-3-morpholino-1-propanol
RLU: Relative light units
RPMI: Roswell Park Memorial Institute
scFv: Single chain variable fragment
SDS-PAGE: Sodium dodecyl sulfate–polyacrylamide gel electrophoresis
SPAAC: Strain-promoted azide-alkyne cycloaddition
Stx1B: Shiga toxin 1 B-subunit
TAA: Tumor-associated antigen
TACA: Tumor-associated carbohydrate antigen
TCR: T cell receptor
TCO: *trans*-Cyclooctene
TME: Tumor microenvironment
Tz: Methyltetrazine
