# Supplementary material for: A bispecific, crosslinking lectibody activates cytotoxic T cells and induces cancer cell death
Source: J Transl Med. 2022 Dec 9;20:578. doi: 10.1186/s12967-022-03794-w (PMC9733292; doi:10.1186/s12967-022-03794-w)
Supplement: Supplementary file 1 — Additional file 1: Figure S1. Expression, purification and characterization of Stx1B K9AzK. Coomassie stained SDS gels showed. a Expression of Stx1B K9AzK with the addition of AzK at a final concentration of 5 mM during induction with IPTG. b No expression of the target protein was detected when the culture medium was not supplemented with AzK during induction. Expression of Stx1B K9AzK with the addition of AzK at a final concentration of 5 mM during induction. M, molecular weight marker, the sizes of the marker bands are indicated; lane 1, clarified lysate; lane 2, flow through after loading clarified lysate on a zinc-charged sepharose column; lane 3, column wash; lanes 4 to 11, elution fractions. c 1.5 µg purified Stx1B K9AzK were subjected to native PAGE, a silver-stained 3–12% PA gel is shown. M, native PAGE marker, marker band sizes indicated in kDa; lane 1, Stx1B K9AzK (MWcalc ~ 45 kDa) in its native pentamer conformation. The black arrows indicate the protein bands of interest. Figure S2. Electrospray ionization mass spectrometry (ESI–MS) measurements of intact a Stx1B, b Stx1B K9AzK, c scFv OKT3 and d scFv OKT3 E129AzK. The proteins identified from the peaks are detailed in the Additional file 1: Table S1. Figure S3. Isothermal titration calorimetry (ITC) analysis of the interaction of a Stx1B and b Stx1B K9AzK with globotriaose. Raw data (top panel) and the binding isotherms obtained by plotting integrated data from titrations (bottom panel) are shown. Figure S4. Size analysis of the Stx1B-scFv OKT3 conjugate. a Representative size exclusion chromatogram of the IEDDA reaction mixture. b Protein standards aprotinin (Apr)- 6500 Da, ovalbumin (O)- 43000 Da, conalbumin (C)- 75000 Da, aldolase (Ald)- 158000 Da and ferritin (F)- 440000 Da were run on the S200 Increase 10/300 GL column for calibration. The column void volume (V0) 7.88 mL was determined by Blue dextran 2000. The gel-phase distribution coefficient (Kav) was calculated using the formula Kav = (Ve-Vo) [file 12967_2022_3794_MOESM1_ESM.docx]

A bispecific, crosslinking lectibody activates cytotoxic T cells and induces cancer cell death

Francesca Rosato^‡1,2^, Rajeev Pasupuleti^‡3,4^, Jana Tomisch^1,2^, Ana Valeria Meléndez^1,2,5^, Dajana Kolanovic^3,4^, Olga N. Makshakova^1,6^, Birgit Wiltschi^3,4,7^,* and Winfried Römer^1,2,8^,*

[1] Faculty of Biology, University of Freiburg, Freiburg, Germany.

[2] Signalling Research Centres BIOSS and CIBSS, University of Freiburg, Freiburg, Germany.

[3] ACIB – The Austrian Centre of Industrial Biotechnology, Graz, Austria.

[4] Institute of Molecular Biotechnology, Graz University of Technology, Graz, Austria.

[5] Spemann Graduate School of Biology and Medicine, University of Freiburg, Freiburg, Germany.

[6] Kazan Institute for Biochemistry and Biophysics, FRC Kazan Scientific Center of RAS, Kazan, Russian Federation.

[7] Institute of Bioprocess Science and Engineering, University of Natural Resources and Life Sciences, Vienna, Austria.

[8] Freiburg Institute for Advanced Studies (FRIAS), University of Freiburg, Freiburg, Germany.

‡ These authors contributed equally to this work

* Corresponding authors:

Email addresses: [winfried.roemer@bioss.uni-freiburg.de](mailto:winfried.roemer@bioss.uni-freiburg.de) (WR); [birgit.wiltschi@acib.at](mailto:birgit.wiltschi@acib.at) (BW)

ORCID iDs: 0000-0002-9255-9391 (Francesca Rosato), 0000-0002-9399-2699 (Rajeev Pasupuleti), 0000-0002-4656-9345 (Jana Tomisch), 0000-0001-6267-2855 (Ana Valeria Meléndez), 0000-0002-2536-1182 (Dajana Kolanovic), 0000-0002-0615-3513 (Olga N. Makshakova), 0000-0001-5230-0951 (Birgit Wiltschi), 0000-0002-2847-246X (Winfried Römer).

**Additional file 1**

**
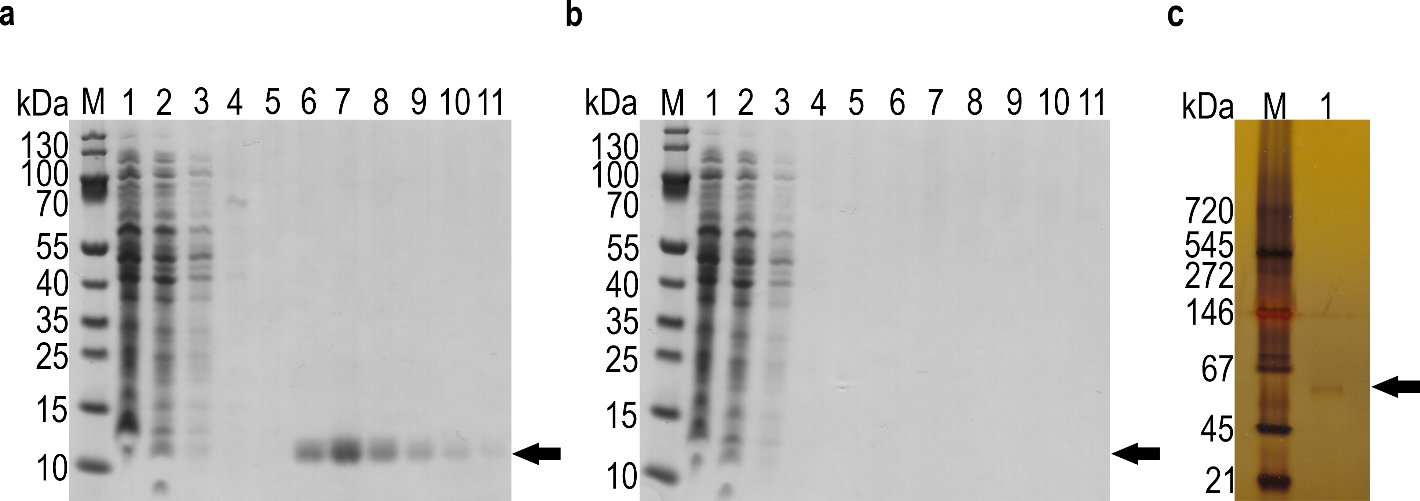
**

**Fig. S1.** Expression, purification and characterization of Stx1B K9AzK. Coomassie stained SDS gels showed. **a** Expression of Stx1B K9AzK with the addition of AzK at a final concentration of 5 mM during induction with IPTG. **b** No expression of the target protein was detected when the culture medium was not supplemented with AzK during induction. Expression of Stx1B K9AzK with the addition of AzK at a final concentration of 5 mM during induction. M, molecular weight marker, the sizes of the marker bands are indicated; lane 1, clarified lysate; lane 2, flow through after loading clarified lysate on a zinc-charged sepharose column; lane 3, column wash; lanes 4 to 11, elution fractions. **c** 1.5 µg purified Stx1B K9AzK were subjected to native PAGE, a silver-stained 3-12% PA gel is shown. M, native PAGE marker, marker band sizes indicated in kDa; lane 1, Stx1B K9AzK (MW_calc_~45 kDa) in its native pentamer conformation. The black arrows indicate the protein bands of interest


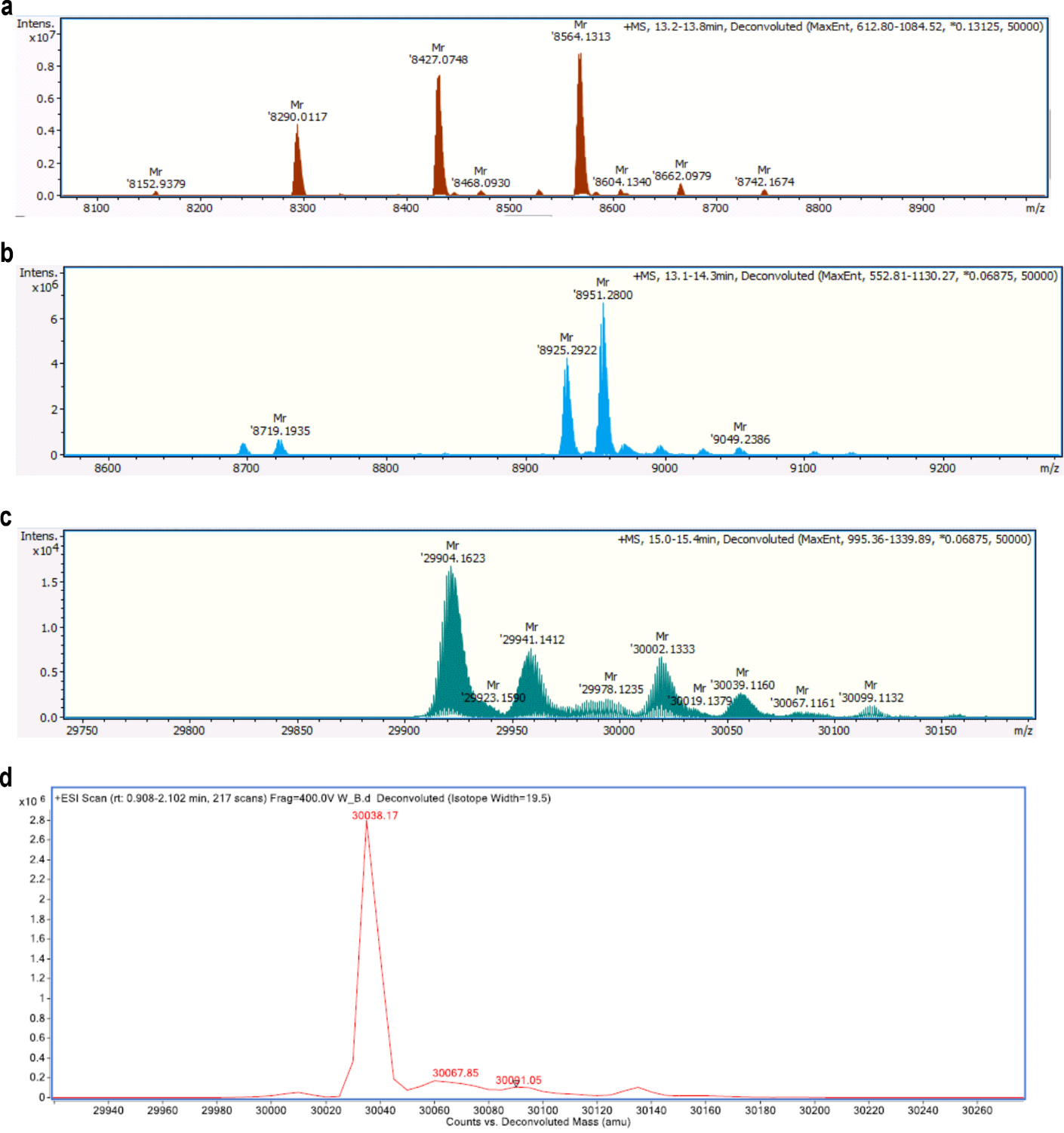


**Fig. S2.** Electrospray ionization mass spectrometry (ESI-MS) measurements of intact **a** Stx1B, **b** Stx1B K9AzK, **c** scFv OKT3 and **d** scFv OKT3 E129AzK. The proteins identified from the peaks are detailed in the Table S1


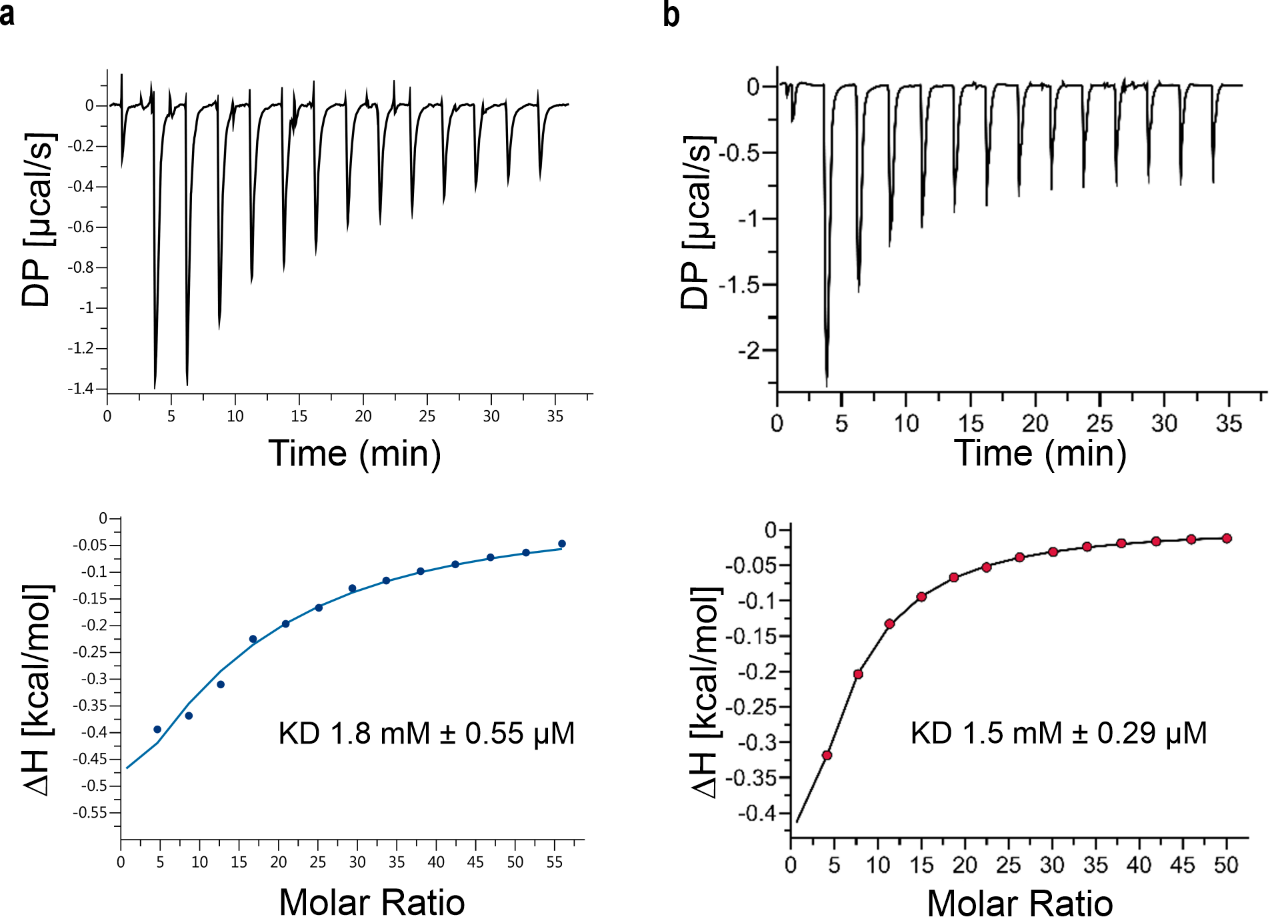


**Fig. S3.** Isothermal titration calorimetry (ITC) analysis of the interaction of **a** Stx1B and **b** Stx1B K9AzK with globotriaose. Raw data (top panel) and the binding isotherms obtained by plotting integrated data from titrations (bottom panel) are shown


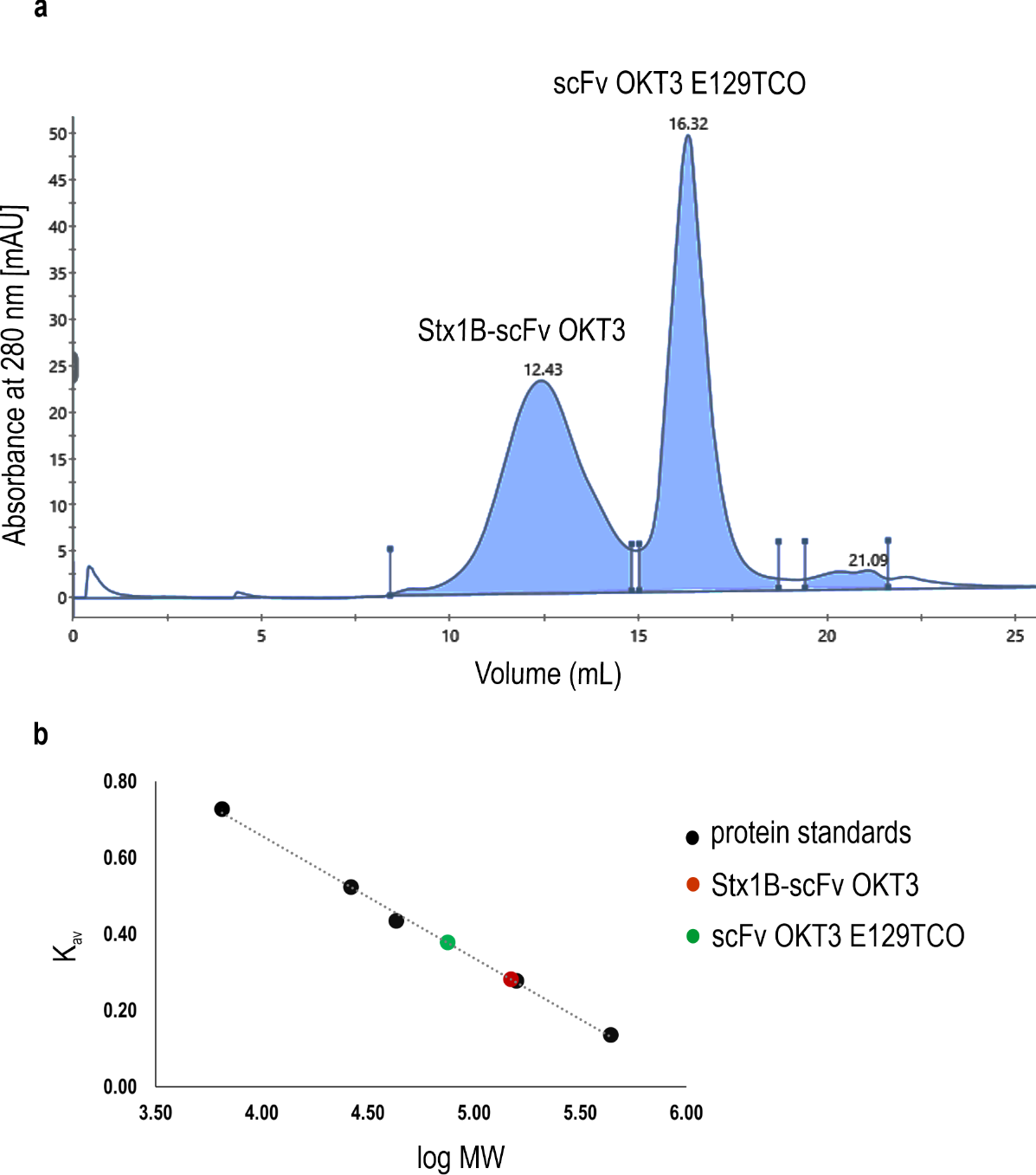


**Fig. S4.** Size analysis of the Stx1B-scFv OKT3 conjugate. **a** Representative size exclusion chromatogram of the IEDDA reaction mixture. **b**Protein standards aprotinin (Apr)- 6500 Da, ovalbumin (O)- 43000 Da, conalbumin (C)- 75000 Da, aldolase (Ald)- 158000 Da and ferritin (F)- 440000 Da were run on the S200 Increase 10/300 GL column for calibration. The column void volume (V_0_) 7.88 mL was determined by Blue dextran 2000. The gel-phase distribution coefficient (*K_av_*) was calculated using the formula *K_av_=(V_e_-V_o_)/(V_b_-V_o_)* where *V_e_* is elution volume and *V_b_* is the column bed volume (24 mL). A calibration curve was plotted with *K_av_* versus the logarithm of molecular weight. The calibration curve calculated from molecular weight standards is a straight line with a coefficient of determination (R^2^) of 0.9972. The red circle represents the Stx1B-scFv OKT3 conjugate and the green circle unreacted scFv OKT3 E129TCO. The straight-line equation K_av_= (-0.3208) x (MW)+1.9415 deduced from the calibration curve was used to determine the experimental molecular weights of the unknown as stated in Table S3

**
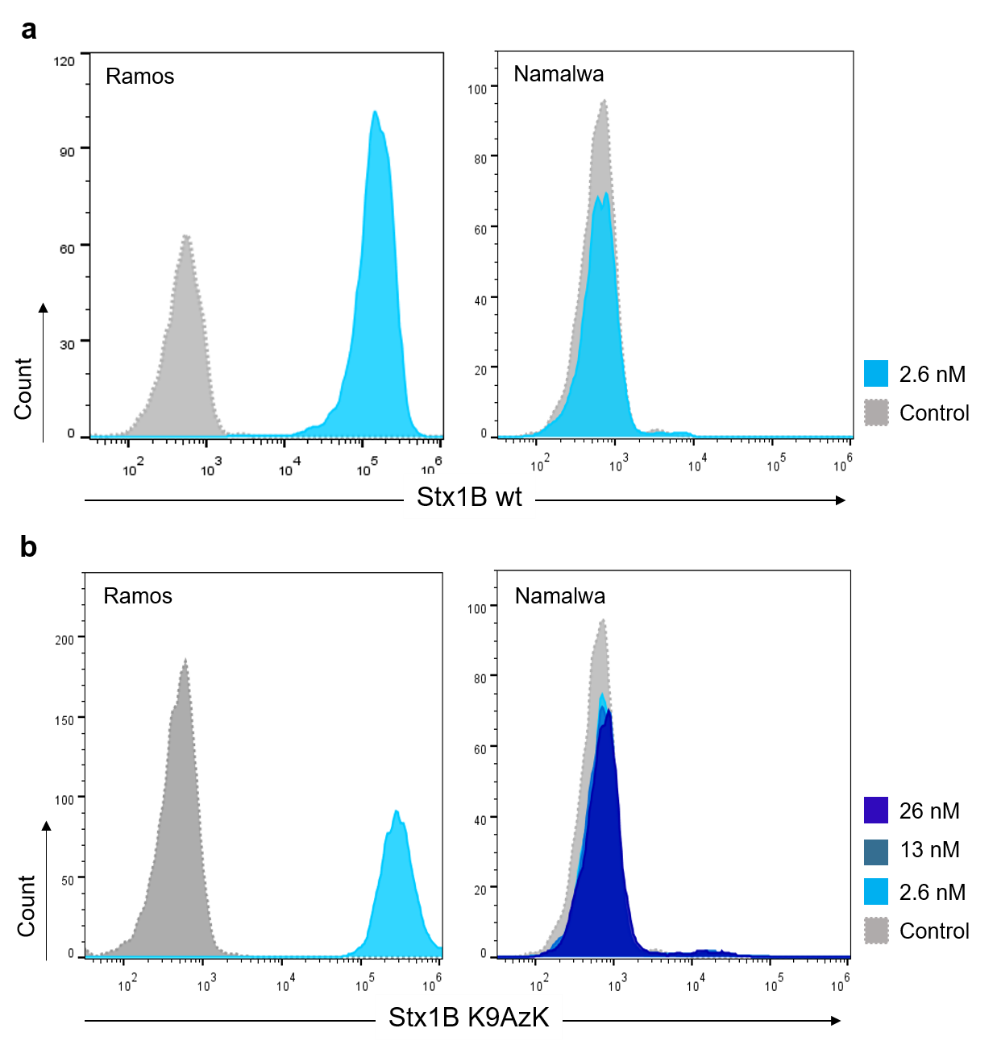
**

**Fig. S5.** Comparative analysis of Stx1B wild-type and mutant proteins binding to tumor cells. Representative histograms of flow cytometry analysis of gated living Ramos and Namalwa cells, incubated with **a** Stx1B wild-type or **b** Stx1B K9AzK for 30 minutes on ice. **a** Histograms of fluorescence intensity of Gb3^+^ Ramos and Gb3⁻ Namalwa cells incubated with Stx1B wt produced in this study (dotted, gray: negative control; light blue: 2.6 nM). **b** Histograms of fluorescence intensity of Ramos and Namalwa cells incubated with Stx1B K9AzK (dotted, gray: negative control; light blue: 2.6 nM; blue: 13 nM; dark blue: 26 nM). Stx1B K9AzK exhibited a similar binding pattern to Gb3 in comparison to the wild-type protein at the cell surface of Gb3^+^ Ramos cells. Fluorescence intensity did not change following incubation of AzK-incorporating Stx1B with Gb3⁻ cells, excluding unspecific binding of the protein to the cell surface, even at higher Stx1B K9AzK concentrations (13 nM and 26 nM). All treated cells were stained with anti-6-His epitope tag AF647 antibody to detect the presence of wild-type and mutant proteins at the plasma membrane. The number of cells within the live population (y-axis) is plotted against the fluorescence intensity of tested proteins (x-axis)


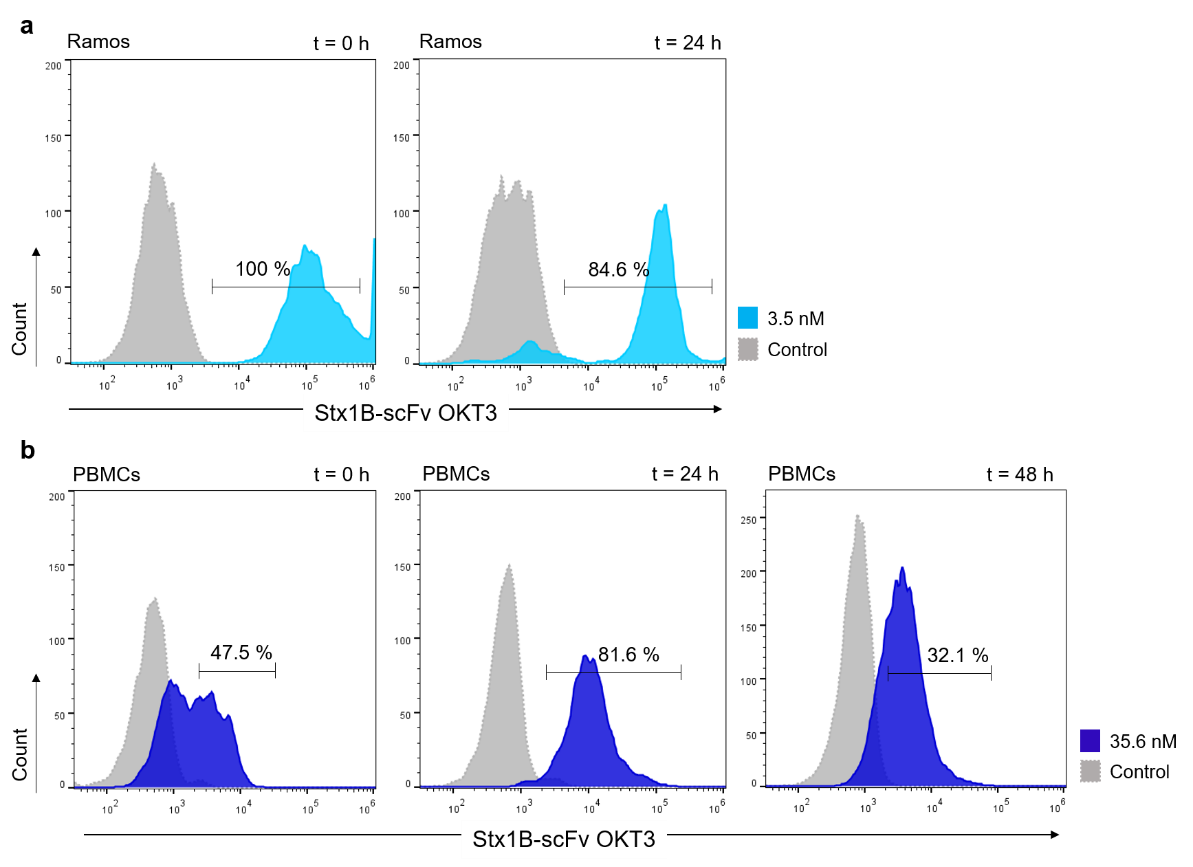


**Fig. S6.** Binding of the Stx1B-scFv OKT3 lectibody to tumor and effector cells after 24 and 48 hours co-incubation. Representative histograms of flow cytometry analysis of gated living **a** Ramos, and **b** PBMCs from healthy donors co-incubated in presence of Stx1B-scFv OKT3 for 30 minutes on ice (t = 0 h) or for 24 and 48 hours at 37°C (t = 24 h, t = 48 h). **a** Histograms of fluorescence intensity of Gb3^+^ Ramos incubated with Stx1B-scFv OKT3 for different time points (dotted, gray: negative control; light blue: 3.5 nM). Histograms depict a stable lectibody binding to Ramos cells after 24 hours (right plot). **b** Histograms of fluorescence intensity of PBMCs incubated with Stx1B-scFv OKT3 for different time points (dotted, gray: negative control; blue: 35.6 nM). Histograms display lectibody binding to CD3 receptors on treated cells. At t = 24 h and t = 48 h following incubation with Gb3^+^ Ramos and Stx1B-scFv OKT3, the lectibody could still be partly detected at the membrane of effector cells. The number of cells within the live population (y-axis) is plotted against the fluorescence intensity of Stx1B-scFv OKT3 (x-axis)

**
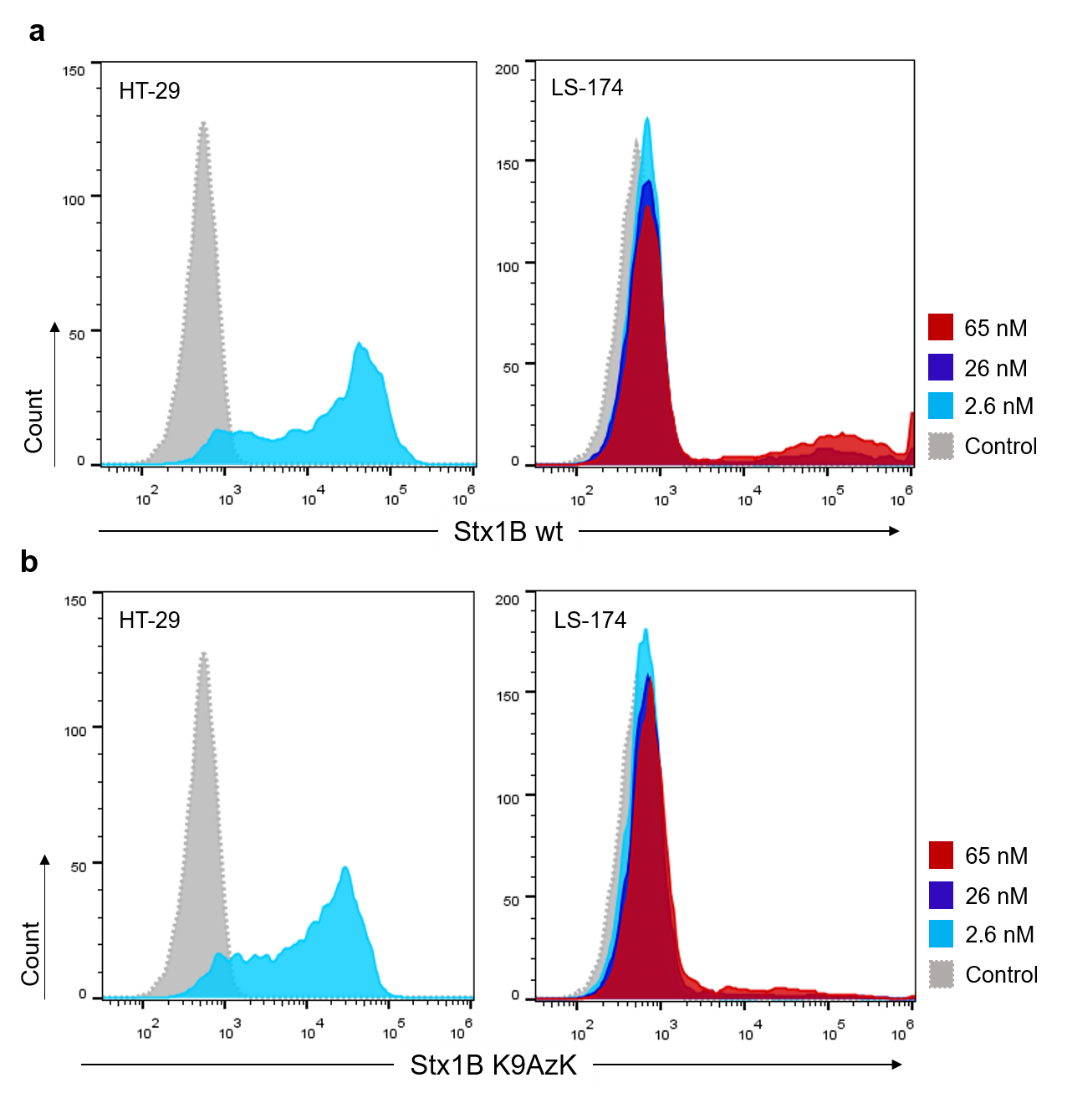
**

**Fig. S7.** Comparative analysis of Stx1B wild-type and mutant proteins binding to solid tumor cells. Representative histograms of flow cytometry analysis of gated living HT-29 and LS-174 cells, incubated with **a** Stx1B wild-type or **b** Stx1B K9AzK for 30 minutes on ice. **a** Histograms of fluorescence intensity of HT-29 and LS-174 cells incubated with Stx1B wt produced in this study (dotted, gray: negative control; light blue: 2.6 nM; blue: 26 nM, red: 65 nM). **b** Histograms of fluorescence intensity of HT-29 and LS-174 cells incubated with Stx1B K9AzK (dotted, gray: negative control; light blue: 2.6 nM; blue: 26 nM; red: 65 nM). Stx1B K9AzK exhibited a similar binding pattern to Gb3 in comparison to the wild-type protein at the cell surface of HT-29 and LS-174 cells. All treated cells were stained with anti-6-His epitope tag AF647 antibody to detect the presence of wild-type and mutant proteins at the plasma membrane. The number of cells within the live population (y-axis) is plotted against the fluorescence intensity of tested proteins (x-axis)

**
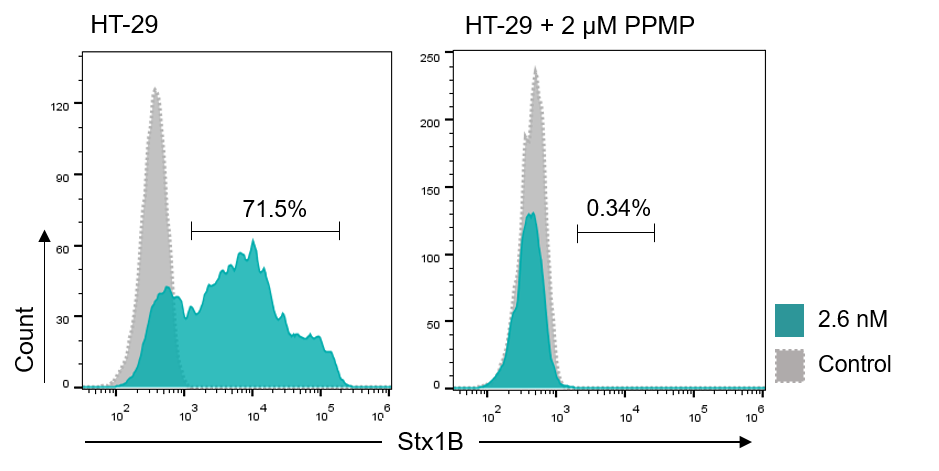
**

**Fig. S8.** Flow cytometry analysis of PPMP-treated HT-29 cells. Representative histograms of gated living HT-29 cells incubated with Stx1B-Cy5 for 30 minutes on ice without (left panel) or after (right panel) Gb3 depletion. At 72 hours post-treatment with 2 µM PPMP, Stx1B no longer bound to HT-29 cells confirming Gb3 depletion from the cell surface

**Tables**

### Table S1.

Analysis of ESI-MS spectral data. Theoretical molecular weights calculated using massXpert [142] were compared to the observed molecular weights of the protein samples. The difference in molecular weight was derived from subtracting observed mass from calculated mass in Daltons; NA, not applicable.

| **Figure no.** | **Sample** | **Calculated mass (Da)** | **Observed mass (Da)** | **Difference in mass (Da)** | **Remarks** |
| --- | --- | --- | --- | --- | --- |
| Fig. S2a | Stx1B-4xH | 8564.1263 | 8564.1313 | -0.0050 | 2xH cleaved during analysis |
| Fig. S2a | Stx1B-3xH | 8427.0674 | 8427.0748 | -0.0074 | 3xH cleaved during analysis |
| Fig. S2a | Stx1B-2xH | 8290.0085 | 8290.0117 | -0.0032 | 4xH cleaved during analysis |
| Fig. S2b | Stx1B K9AzK-6xH | 8952.27 | 8951.28 | 0.99 | AzK incorporation |
| Fig. S2c | scFv OKT3-6xH | 29904.1160 | 29904.1623 | -0.0463 | NA |
| Fig. S2d | scFv OKT3 E129AzK-6xH | 30035.88 | 30038.17 | -2.29 | 2x disulfide bonds |

| **Figure no.** | **Sample** | **Calculated mass (Da)** | **Observed mass (Da)** | **Difference in mass (Da)** | **Remarks** |
| --- | --- | --- | --- | --- | --- |
| Fig. S2a | Stx1B-4xH | 8564.1263 | 8564.1313 | -0.0050 | 2xH cleaved during analysis |
| Fig. S2a | Stx1B-3xH | 8427.0674 | 8427.0748 | -0.0074 | 3xH cleaved during analysis |
| Fig. S2a | Stx1B-2xH | 8290.0085 | 8290.0117 | -0.0032 | 4xH cleaved during analysis |
| Fig. S2b | Stx1B K9AzK-6xH | 8952.27 | 8951.28 | 0.99 | AzK incorporation |
| Fig. S2c | scFv OKT3-6xH | 29904.1160 | 29904.1623 | -0.0463 | NA |
| Fig. S2d | scFv OKT3 E129AzK-6xH | 30035.88 | 30038.17 | -2.29 | 2x disulfide bonds |

### Table S2.

Stx1B-scFv OKT3 conjugate identified by mass analysis. The in-gel tryptic digest of the 50 kDa band from lane 4, Fig 3b confirmed peptides corresponding to scFv OKT3 and Stx1B in the Stx1B-scFv OKT3 conjugate. ID, Identity; Da, Daltons.

| **Sample** | **Protein IDs** | **Peptide sequence** | **Observed mass (Da) of the peptide** |
| --- | --- | --- | --- |
| 50 kDa band (lane 4, Fig 3b) | scFv OKT3 | ASGYTFTR | 901.42938 |
|  |  | ATLTTDKSSSTAYMQLSSLTSEDSAVYYCAR | 3422.5494 |
|  |  | GYTNYNQK | 986.44576 |
|  |  | LASGVPAHFR | 1053.572 |
|  |  | LEINRADTAPTGSEQK | 1728.8642 |
|  |  | MSCKASGYTFTR | 1423.6224 |
|  |  | PGQGLEWIGYINPSR | 1685.8526 |
|  |  | QRPGQGLEWIGYINPSR | 1970.0122 |
|  |  | SSSTAYMQLSSLTSEDSAVYYCAR | 2692.1633 |
|  |  | VDIVLTQSPAIMSASPGEK | 1958.003 |
|  |  | VTMTCSASSSVSYMNWYQQK | 2389.0025 |
|  |  | WIYDTSK | 911.43888 |
|  |  | YYDDHYSLDYWGQGTTLTVSSAK | 2669.1922 |
|  | Stx1B | TNACHNGGGFSEVIFR | 1764.8002 |
|  |  | VEYTKYNDDDTFTVK | 1836.8418 |
|  |  | VGDKELFTNR | 1177.6091 |
|  |  | YNDDDTFTVK | 1216.5248 |
|  |  |  |  |

### Table S3.

Experimental molecular weights calculated from the calibration curve shown in Fig. S4. Analysis showed that three scFv OKT3 molecules were conjugated to one Stx1B pentamer. Proteins eluted from two peaks in SEC (Fig. S4) correspond to Stx1B-scFv OKT3 conjugate and unreacted scFv OKT3 E129TCO. Theoretical molecular weights were calculated using Expasy ProtParam. NA, not applicable

| **sample type** | **sample name** | **log MW** | **Kav** | **Theoretical MW (Da)** | **Experimental MW (Da)** |
| --- | --- | --- | --- | --- | --- |
| standard | aprotinin | 3.81 | 0.72 | 6500 | NA |
| standard | ovalbumin | 4.68 | 0.43 | 43000 | NA |
| standard | conalbumin | 4.87 | 0.37 | 75000 | NA |
| standard | aldolase | 5.19 | 0.27 | 158000 | NA |
| standard | ferritin | 5.64 | 0.13 | 440000 | NA |
| sample | scFv OKT3 E129TCO | 4.41 | 0.52 | 29927 | 26300 |
| sample | Stx1B pentamer + 3 molecules of scFv OKT3 + linkers | 5.17 | 0.28 | 139456 | 149325 |
